# Altering Affective Responses and Emotional Reactivity Through Acute Bouts of Aerobic Exercise With and Without Mindfulness

**DOI:** 10.1111/psyp.70405

**Published:** 2026-09-25

**Authors:** Salim Onbasi, Wojciech Kielbus, C. J. Brush, Steve Amireault, Sarah Ullrich‐French, Yu‐Kai Chang, Shih‐Chun Kao

**Affiliations:** ^1^ Department of Health and Kinesiology Purdue University West Lafayette Indiana USA; ^2^ School of Kinesiology Auburn University Auburn Alabama USA; ^3^ Department of Kinesiology and Educational Psychology Washington State University Pullman Washington USA; ^4^ Department of Physical Education National Taiwan Normal University Taipei Taiwan

**Keywords:** affect, emotional regulation, event‐related potential, LPP, physical activity

## Abstract

Although the beneficial effects of acute aerobic exercise (AE) and mindfulness (MF) on emotional well‐being are well‐documented, whether these two intervention modalities can be combined to maximize these benefits remains unclear. The purpose of this study was to investigate the combined effect of AE and MF on affective responses and emotional reactivity. Using a within‐subject crossover design, self‐reported affect was assessed from 30 adults (15 male) aged 18–25 before and after a 30 min session of mindful AE (MF‐AE), non‐mindful AE (NM‐AE), and seated rest on three separate days in counterbalanced order. Following each intervention, emotional reactivity was measured by the late positive potential (LPP) of event‐related potential (ERP) during a computerized picture‐viewing task consisting of neutral and pleasant/unpleasant emotional content. Analysis indicated no between‐condition differences in affect at the pretest, while there was increased positive affect for MF‐AE and NM‐AE conditions compared with rest at posttest. Although LPP amplitudes did not differ between conditions, greater negative (LPP_unpleasant_–LPP_neutral_) compared to positive (LPP_pleasant_–LPP_neutral_) emotional reactivity was observed with small‐to‐moderate and moderate‐to‐larger effect sizes following the NM‐AE and rest conditions, respectively. However, this reactivity‐related difference was absent following the MF‐AE condition. Subsequent analyses indicated reduced dominance of negative reactivity during both the early (400–1200 ms) and later (1200–2000 ms) post‐stimulus windows following the MF‐AE condition, while this mitigation effect emerged only during the later window following the NM‐AE condition. In addition to the AE‐induced acute benefits to self‐reported affective outcomes, performing MF during AE may accelerate the elimination of the tendency in reacting to negative emotion‐eliciting stimuli.

## Impact Statement

This study demonstrates that aerobic exercise with and without simultaneous mindfulness both transiently induce a positive effect on affect. Aerobic exercise combined with mindfulness reduced neural reactivity to negative emotional stimuli, while a similar mitigation effect was observed only during the later emotional processing stage following aerobic exercise alone, suggesting a temporally additive benefit from aerobic exercise and mindfulness to neural processing underlying emotional regulation. These findings highlight mindful aerobic exercise as a promising strategy for improving emotion and inform the development of more effective, integrative mind–body interventions for mental health promotion.

## 1 Introduction

Emotion and its regulation play vital roles to support academic achievement (Pekrun 2017) and positive work outcomes (Chandra et al. 2020) as well as mitigating cardiovascular health risk (DeSteno et al. 2013) and problem behaviors (Roberton et al. 2012). However, the fluctuating nature of emotions makes young individuals during adolescence and emerging adulthood particularly vulnerable because of rapid hormonal and neurodevelopmental changes (Liew et al. 2023) and unstable biological, social, or environmental resources available for coping with stress (Zimmermann and Iwanski 2014). The vulnerability during this developmental period can be further exacerbated by sedentary lifestyles that are associated with physical inactivity (Isasi et al. 2013) and non‐cognitively engaging screen time (Twenge and Campbell 2018). There is a critical need to identify strategies for improving emotional well‐being by targeting physical activity and cognitive engagement.

Interventions delivering physical and cognitively engaging activities such as aerobic exercise (AE) (Reed and Ones 2006; Reed and Buck 2009; Ekkekakis and Petruzzello 1999) and mindfulness (MF) (Schumer et al. 2018; Sedlmeier et al. 2012) have demonstrated efficacy in improving emotion. According to systematic reviews and meta‐analyses, chronic and acute engagement in AE (Reed and Ones 2006; Reed and Buck 2009; Ekkekakis and Petruzzello 1999) and MF (Schumer et al. 2018; Sedlmeier et al. 2012) have both been linked to improved affect and mood. These positive effects, as part of the larger encompassing social cognitive benefits (Ludyga et al. 2022), of AE have been linked with various mechanisms at the neuromolecular level (e.g., neurotrophic factors, serotonin and norepinephrine, regulation of hypothalamic–pituitary–adrenal axis activity), cerebral vasculature, and structural and functional brain adaptation in frontal cortex, hippocampus, and frontal‐limbic neural networks (Gujral et al. 2017; Basso and Suzuki 2017). On the other hand, MF has been linked to improved emotional outcomes (Hill and Updegraff 2012; Grecucci et al. 2015; Leyland et al. 2019; Arch and Craske 2006; Farb et al. 2010) through enabling the control of attention at the present in a nonjudgmental manner, the acquisition of attentional regulation and meta‐cognitive skills, as well as underlying neuroplastic changes in the anterior cingulate cortex, insula, temporo‐parietal junction, frontolimbic network, and default mode network structures (Bishop et al. 2004; Hölzel et al. 2011). Of these potential mechanisms, emotional reactivity (Brush et al. 2020, 2021; Ligeza et al. 2023; Lin et al. 2016, 2024; Uusberg et al. 2016), an early perceptual and automatic process biasing attention in facilitating the subsequent emotional processing, has emerged as a neurocognitive mechanism to reveal how AE and MF differentially influence the generation and regulation of emotion.

Through recording event‐related potentials (ERPs) derived from electroencephalogram (EEG), emotional reactivity can be quantified during tasks involving viewing of images with varying levels of arousal and valence. Specifically, the late positive potential (LPP) is elicited by emotionally evocative content, beginning around 300–400 ms after stimulus onset (Hajcak et al. 2010) and persisting throughout stimulus presentation duration (Cuthbert et al. 2000). The LPP is indicative of motivated attentional engagement to stimulus significance (Hajcak and Foti 2020) and is thought to be modulated by the activation of cortico‐limbic appetitive and aversive motivational systems (Bradley 2009) as well as cortical and subcortical neural structures implicated in emotional processing (Sabatinelli et al. 2013). Interpretations of the LPP center on its amplitude, with amplitudes increasing in response to pleasant and unpleasant images relative to neutral images (Hajcak and Foti 2020), indicating sustained and potentiated attentional engagement during viewing of emotional stimuli (Brush et al. 2025). To assess emotional reactivity, researchers use a difference wave approach to isolate brain activity unique to emotional content, wherein the neural responses to neutral images are subtracted from the neural responses to pleasant/unpleasant images.

Research examining the LPP during passive picture‐viewing paradigms has shown that a single session of AE enhances LPP amplitude, particularly for positive emotional reactivity (i.e., increased neural activity to positive emotion‐eliciting images) (Brush et al. 2020, 2021; Ligeza et al. 2023), while diminished negative emotional reactivity, as indexed by attenuated differences in LPP between neutral and negative emotion‐eliciting events, has been observed following MF (Lin et al. 2016, 2024; Uusberg et al. 2016). Despite these promising results and the increasing interest in combining AE and MF for optimal emotional benefits, AE and MF have usually been delivered on different days within a program (Zhang et al. 2019; Schneider et al. 2019) or at different times within a session (Edwards and Loprinzi 2019). Although recent studies have begun exploring the effect of MF concurrently during AE on self‐reported affect, pleasure, and enjoyment (Cox et al. 2020, 2018; Ullrich‐French et al. 2024), whether this integrated approach can be applied to optimize emotional reactivity by enhancing positive emotional reactivity and reducing negative emotional reactivity remains unknown.

MF during exercise can lead to positive affective responses, likely because mindfully regulating breathing and attentional focus is positively related to exercise performance (e.g., time, speed, strength, accuracy, balance) (Laborde et al. 2022; Kee et al. 2012) and experiences (e.g., physical concept, reasons to exercise) (Laborde et al. 2021; Cox et al. 2016). Further, exercise‐induced psychophysiological changes could amplify the somatic responses and thought processes to be sensed and regulated (Smirmaul 2010; Gibson and Foster 2007; Gammage et al. 2001), facilitating the process of achieving a more mindful state. Such a virtuous circle by performing AE concurrently with MF, compared with AE alone, makes this mind–body approach particularly meaningful as it can be theorized to have greater benefits to state‐related affect and potentially the subsequent formation of positive memory traces to influence future decisions to engage in or adhere to exercise (Ekkekakis and Brand 2019). Practically, the multimodal approach delivering two emotion‐enhancing behaviors together and engaging exercisers' minds during physical activity enables the maximization of intervention efficiency and acceptability while complementing the efforts in promoting mental health through mindful physical activity (Lawlor 2016; Jones and Bouffard 2012).

The purpose of this study was to employ a novel mindful AE (MF‐AE) intervention protocol that delivered MF and AE in tandem to enhance emotional reactivity. A randomized cross‐over design was used to compare neural emotional reactivity between the MF‐AE, non‐mindful AE (NM‐AE), and rest intervention conditions. It was hypothesized that both MF‐AE and NM‐AE would lead to increased positive emotional reactivity (e.g., increased LPP amplitude in response to pleasant images) (Brush et al. 2020, 2021; Ligeza et al. 2023) than rest control while MF‐AE would lead to additional reduction of negative emotional reactivity (e.g., decreased LPP amplitude in response to unpleasant images) (Lin et al. 2016, 2024; Uusberg et al. 2016). Given the previous work showing beneficial effects of AE on affect, enjoyment, and pleasure, these were also included as secondary emotional outcomes to test the hypothesis that MF‐AE and NM‐AE would lead to more positive affect and greater enjoyment and pleasure compared with the rest control, while the benefits from MF‐AE would be greater compared with NM‐AE.

## 2 Methods

### 2.1 Participants

Young adults aged 18–25 from a Midwest college town in the United States were recruited to complete three laboratory visits. Participants were included if they self‐reported being free from diagnosed medical conditions that would preclude participation in exercise (Physical Activity Readiness Questionnaire) (Thomas et al. 1992), no cognitive or physical disabilities, no medications for attention‐deficit/hyperactivity disorder, normal or corrected‐to‐normal vision, and proficient in English. The 21‐item version of the Depression Anxiety Stress Scale (DASS‐21) was used to assess the presence of internalizing symptoms over the past week, including the depression, anxiety, and stress scales. Participants who scored outside normal ranges on the depression (0–9), anxiety (0–7), and stress (0–14) scales were excluded given the associations between these symptoms and emotional outcomes of this study (i.e., LPP) (Lovibond and Lovibond 1995; Henry and Crawford 2005; Proudfit et al. 2015; MacNamara and Hajcak 2010). All research procedures were approved by the Institutional Review Board at Purdue University and adhered to the Declaration of Helsinki guidelines (protocol number: IRB‐2024‐355). Participants provided written informed consent. Of the 55 screened participants, 32 were eligible and enrolled. Two participants withdrew due to time conflict and loss of interest, resulting in a total of 30 participants included for analysis. With statistical power = 0.80 and *α* = 0.05, sensitivity analysis indicated this sample was sufficient to detect repeated measures ANOVA interactions (*f* = 0.25) and two‐tailed paired *t*‐test difference (Cohen's *d* = 0.54) for acute AE (Brush et al. 2021) or MF (Lin et al. 2016) effects on emotional reactivity.

### 2.2 Intervention Manipulation Assessments

Exercise‐induced state‐mindfulness was measured using the State Mindfulness Scale for Physical Activity 2 (SMS‐PA2) (Ullrich‐French et al. 2022). The SMS‐PA2 included 15 items to assess the monitoring and accepting dimensions and the body and mind focuses within each dimension (Ullrich‐French et al. 2022), including monitoring of the mind (4 items), monitoring of the body (4 items), accepting of the mind (3 items), and accepting of the body (4 items) categories. All items are rated on a 5‐point scale, ranging from 0 (“Not at all”) to 4 (“Very much”). Average scores for each category (internal consistency α and ω ranging from 0.79 to 0.92 and 0.79 to 0.92, respectively) and the sum of these average scores were computed, with higher scores indicating greater levels of category‐specific and global levels of state‐mindfulness. Perceived cognitive exertion was assessed using the single‐item mental effort scale (MES) (Ouwehand et al. 2021) with a 9‐point scale ranging from 1 (“Very, very low mental effort*”*) to 9 (“Very, very high mental effort*”*), while physical exertion was quantified by heart rate using a chest band monitor (Garmin, HRM‐Dual; Olathe, KS).

### 2.3 Affective Assessments

Affective valence was measured using a single‐item feeling scale (FS) (Hardy and Rejeski 1989) with an 11‐point scale ranging from −5 (“Very bad”) to +5 (“Very good”) and a zero indicating “Neutral”. Participants were asked to indicate how they felt in the moment. Remembered pleasure (RP) was measured using a visual analog scale (Zenko et al. 2016) where participants responded to the question, “How did the exercise session in the laboratory make you feel?” by marking a point on a continuum from very pleasant (+100) to very unpleasant (−100). Similarly, predicted pleasure (PP) was assessed with an empirical valence scale through the question, “If you repeated the exercise session, how do you think it would make you feel?” Participants marked their responses on a scale that included 15 empirically spaced verbal anchors which ranged from the most unpleasant imaginable (−100) to the most pleasant imaginable (+100) (Zenko et al. 2016). Verbal descriptors on the RP and PP scales were visible to participants, while the numerical values were only used for scoring, with higher RP and PP scores indicating more positive remembered and predicted pleasure, respectively. The Physical Activity Enjoyment Scale (Kendzierski and DeCarlo 1991) (PACES) includes 18 items reflecting feelings and attitudes toward physical activity and was used to assess exercise‐related enjoyment. The respondents were instructed to indicate “how you feel at the moment about the physical activity you have been doing.” Participants rated each item (e.g., “I enjoy it” versus “I hate it”) on a 7‐point scale, with a higher total score indicating greater enjoyment of physical activity (internal consistency *α* = 0.93 and *ω* = 0.93).

### 2.4 Emotional Reactivity Assessment

Emotional processes were elicited by a picture‐viewing task using visual stimuli sourced from the International Affective Picture System (IAPS) (Lang et al. 2005). A total of 126 images (see supplement for image numbers), including 42 pleasant (e.g., affectionate couples, infants, household items), 42 neutral (e.g., architectural structures, neutral facial expressions), and 42 unpleasant images (e.g., injuries, violent scenarios), were arranged into three blocks. Each block consisted of 14 pleasant, 14 neutral, and 14 unpleasant images that were presented in random order. Each image was presented full screen in color for 2000 ms, with a white fixation cross shown focally during a variable intertrial interval ranging from 1700 to 2300 ms after stimulus onset. Images of each category included in the three blocks were matched using the norm‐based self‐assessment manikin (Bradley and Lang 1994) (SAM) ratings of valence (Block 1: neutral = 5.01 ± 0.35; pleasant = 7.29 ± 0.61; unpleasant = 2.47 ± 0.81; Block 2: neutral = 5.01 ± 0.20; pleasant = 7.21 ± 0.55; unpleasant = 2.46 ± 0.73; Block 3: neutral = 5.02 ± 0.31; pleasant = 7.25 ± 0.52; unpleasant = 2.45 ± 0.75) and arousal (Block 1: neutral = 2.83 ± 0.69; pleasant = 5.08 ± 0.54; unpleasant = 6.21 ± 0.48; Block 2: neutral = 2.81 ± 0.40; pleasant = 5.04 ± 0.67; unpleasant = 6.21 ± 0.48; Block 3: neutral = 2.82 ± 0.50; pleasant = 5.07 ± 0.66; unpleasant = 6.18 ± 0.55). In the normed ratings, the valence scale included five graphic depictions representing a continuum from unhappy to happy, with numbers 1 through 9 displayed below the depictions, accompanied by an explanation that includes examples of relevant emotional experiences such as panic, disgust, despair, as well as fun, happiness, and satisfaction. The arousal scale also included five graphic depictions that convey a range of visceral reactions from calm to excited. Accompanying these illustrations are numbers 1 to 9, with 1 indicating no bodily response at all and 9 reflecting a strong bodily response. Below the graphic depiction, an explanation is provided with examples of related states such as relaxation, tranquility, excitation or excitement, and anger (Lang et al. 2005; Weinberg and Hajcak 2010).

To confirm similar ratings of valence and arousal within the current sample, participants were asked to evaluate each of the 126 images and provide their valence and arousal ratings using SAM following the completion of the rest condition. The sample‐based participant ratings showed the most positive valence rating for pleasant images followed by neutral and unpleasant images across blocks (Block 1: neutral = 5.03 ± 0.21; pleasant = 6.48 ± 0.52; unpleasant = 2.47 ± 0.98; Block 2: neutral = 4.98 ± 0.15; pleasant = 6.63 ± 0.51; unpleasant = 2.62 ± 0.82; Block 3: neutral = 4.98 ± 0.29; pleasant = 6.64 ± 0.41; unpleasant = 2.46 ± 0.86) as well as the highest arousal rating for unpleasant images followed by pleasant and neutral images across blocks (Block 1: neutral = 1.59 ± 0.39; pleasant = 3.33 ± 0.46; unpleasant = 5.27 ± 1.33; Block 2: neutral = 1.40 ± 0.17; pleasant = 3.65 ± 0.65; unpleasant = 5.05 ± 1.21; Block 3: neutral = 1.61 ± 0.29; pleasant = 3.55 ± 0.55; unpleasant = 5.27 ± 1.18). These sample‐based ratings confirmed differences in valence and arousal ratings between image types and matched ratings of valence and arousal across three testing blocks (see supplement for ratings from individual participants in this sample).

### 2.5 EEG Signal Processing and ERP Measurement

During the picture‐viewing task, EEG was recorded from 64 electrodes configured according to the international 10–20 system on a Neuroscan Quikcap (Compumedics, Charlotte, NC). All electrodes maintained an impedance < 10 kΩ prior to the start of recording. The recording was referenced online using CCPz with the AFz electrode serving as the ground. Additional electrodes were placed above and below the left orbit and outer canthus of each eye to monitor electrooculographic (EOG) activity. Continuous data were sampled at a rate of 1000 Hz and amplified by 500 times with a DC to 70 Hz filter and a 60 Hz notch filter using a Neuroscan SynAmps2 amplifier (Compumedics, Charlotte, NC).

**FIGURE 1:**
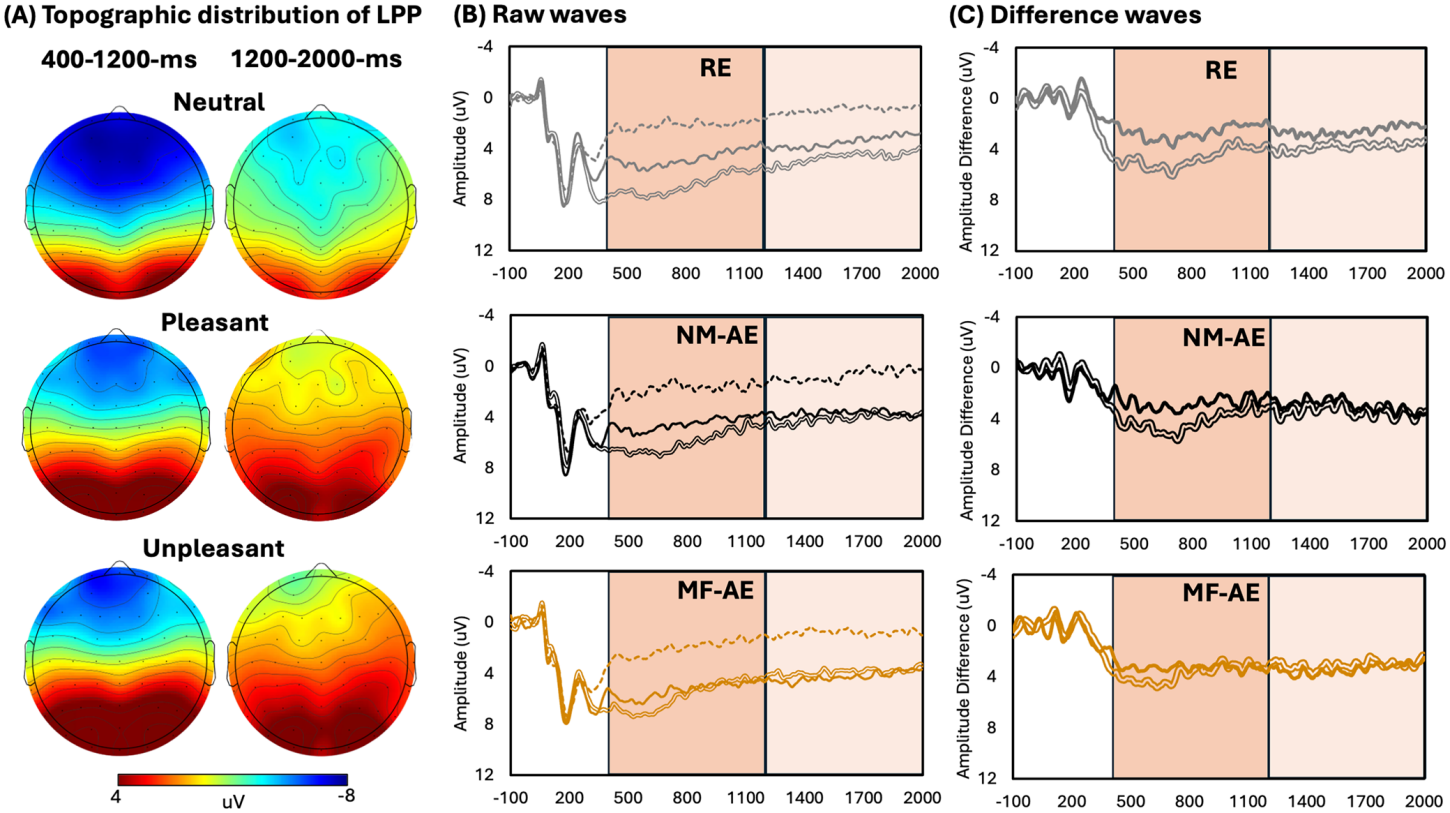
The subplot (A) shows the topographical distribution of LPP amplitudes elicited by neutral, pleasant, and unpleasant images during the early 400–1200 ms and later 1200–2000 ms post‐stimulus windows. The subplot (B) shows raw LPP waveforms of neutral (dashed lines), pleasant (solid lines), and unpleasant (doubled lines) following mindfulness aerobic exercise (MF‐AE), non‐mindful aerobic exercise (NM‐AE), and rest (RE) intervention conditions. The subplot (C) shows the difference waves of positive reactivity (solid lines) and negative reactivity (doubled lines) following three intervention conditions. In the subplots (B) and (C), the 400–1200 ms and 1200–2000 ms post‐stimulus windows are highlighted in dark pink and light pink, respectively. The ERP waveforms were created using the region of interest (ROI) including parieto‐occipital electrode sites (P1, PZ, P2, PO3, POZ, PO4, O1, OZ, and O2).

For offline data processing, MATLAB (R2023a, Mathworks Inc.; Natick, MA), EEGLAB toolbox (version 4.1.1b) (Delorme and Makeig 2004), and ERPLAB toolbox (version 6.1.4) (Lopez‐Calderon and Luck 2014) were utilized. Continuous data were re‐referenced to averaged mastoids (M1, M2). The independent component analysis (ICA) (Pontifex et al. 2017) was used to correct eyeblink artifacts and the corrected data were segmented to generate epochs ranging from −100 to 2000 ms that were aligned with the onset of the stimulus. Baseline correction was applied using the pre‐stimulus interval from −100 to 0 ms. Additionally, a zero phase‐shift bandpass filter (IIR Butterworth filter) with a cutoff range of 0.01 to 30 Hz (24 dB/oct) was applied and EEG epochs were rejected if an identified artifact exceeded ±100 μV or the overall variance of the epoch exceeded ±3 standard deviations of the mean of local (by electrode) and global (all electrodes) accepted epochs. The mean numbers of accepted epochs did not differ between MF‐AE, NM‐AE, and rest conditions for pleasant (32.6 ± 7.6 vs. 33.0 ± 7.5 vs. 33.6 ± 4.1), unpleasant (31.6 ± 8.0 vs. 31.8 ± 7.3 vs. 34.1 ± 3.9), or neutral (32.8 ± 6.3 vs. 32.7 ± 7.7 vs. 34.3 ± 3.9) images (*p*s > 0.24). Grand‐average waves for three image types were computed across intervention conditions to test whether the LPP was modulated by valence. LPP was quantified using a 400–2000 ms post‐stimulus window at the region of interest (ROI), including parieto‐occipital sites (P1, PZ, P2, PO3, POZ, PO4, O1, OZ, and O2) consistent with previous studies (Brush et al. 2020, 2021; Ligeza et al. 2023) and visual inspection of the current grand‐averaged data (Figure 1A). To verify the successful manipulation of valence‐induced differences, analysis using LPP amplitudes was first conducted. For the primary analyses (Brush et al. 2020), subtraction‐based difference waves (LPP_diff_) were constructed by subtracting the LPP to neutral from LPP to pleasant images and subtracting the LPP to neutral from LPP to unpleasant images to isolate neural activity specific to positive and negative emotional reactivity, respectively. Given the apparent difference in raw (Figure 1B) and difference (Figure 1C) waves between the 400–1200 ms and 1200–2000 ms post‐stimulus windows during the stimulus presentation, data‐driven post hoc analysis was conducted to determine intervention effects in the early and later stages of emotional processing, separately.

### 2.6 Intervention Conditions

For the MF‐AE condition, participants completed a 30 min exercise on a treadmill, which included a 2 min warm‐up, 26 min of brisk walking at 64%–76% of age‐predicted (208−[0.7 × age]) maximal heart rate (HR_max_) (Tanaka et al. 2001), and a 2 min cool‐down phase. The treadmill speed was set between 2 and 3 mph with 0% incline during the warm‐up and cool‐down phases. To achieve the target HR during brisk walking, increasing incline was prioritized over speed to prevent running and allow participants to concentrate on the MF cues delivered through an over‐ear wireless headset (Bluedee, BH200, 20 KHz, B0C2D21HBX, 2023) with active noise cancellation features. During the entire 30 min exercise, participants listened to a 30 min audio recording that guided their attention to thoughts, breathing, and body sensations with a focus on the present moment without judgment. The audio was recorded by a female native English speaker in a professional media production room and was designed to be temporally synchronized with the three different intervention phases. Specifically, the 30 min audio included a total of 3, 40, and 3 clusters of cues in the warm‐up, brisk walking, and cool‐down phases, respectively. Each cue cluster included 1–5 sentences to target the body and mind focuses of monitoring and accepting dimensions (Table 1). Due to the nature of AE and its resulting observable changes in various physiological responses and body sensations, the audio naturally included more cues for the monitoring dimension than the accepting dimension as well as more cues for the body focus than the mind focus. Previous research has demonstrated the effectiveness of similar audio recordings in inducing state‐mindfulness while walking on a treadmill (Ullrich‐French et al. 2024).

**TABLE 1:** Sample cues included in the mindfulness audio based on the dimension and focus.

	Cue	Dimension	Focus
Warm‐up	Let's begin by gently drawing attention to the sensations of your breathing. Notice the sensations of breathing wherever you sense it in the body.	Monitoring	Body
Brisk walking	It is normal to notice that your mind is wandering. If you become distracted or feel your mind wandering, gently come back to the sensations in your body. If other feelings come into awareness, simply acknowledge them, and then bring your focus gently back to your breathing in this moment. Take some time now to just feel the sensations of breathing.	Monitoring	Mind
		Accepting	
	Continue to pay attention to the sensations in the body. Without judgment or opinion, just do this with a sense of openness and curiosity as different feelings, sensations, and experiences emerge and change. You may direct your attention to your physical experience in any part of your body. You can start the sensation from your hands, and let the sensation travel to your chest and forehead like before. You can also explore sensations from any other areas in your body	Monitoring	Body
		Accepting	
	Let your forehead be the final traveling destination of the sensation, and use your forehead as a door to allow your thoughts to exit.	Monitoring	Mind
Cool‐down	How does your body feel right now walking on the treadmill? Do you notice any change in your heartbeat or how you breathe? Perhaps you notice intense sensations in parts of your body or perhaps you feel energized and strong. Step back in your mind's eye again and just observe all of the different sensations in your body as they come and go without judging them	Monitoring	Body
		Accepting	

The NM‐AE condition was identical to MF‐AE, except that the 30 min audio was replaced by a 30 min podcast about increasing longevity (https://tinyurl.com/5n7tpsxu). During the rest control, participants were seated while listening to a different 30 min podcast about salt and cardiometabolic health (https://tinyurl.com/3paj349s). These health‐related podcasts were included as part of the NM‐AE and rest conditions to ensure the presence of audio stimuli that were assumed to have a minimum MF‐inducing effect (Ullrich‐French et al. 2024).

### 2.7 Procedure

**FIGURE 2:**
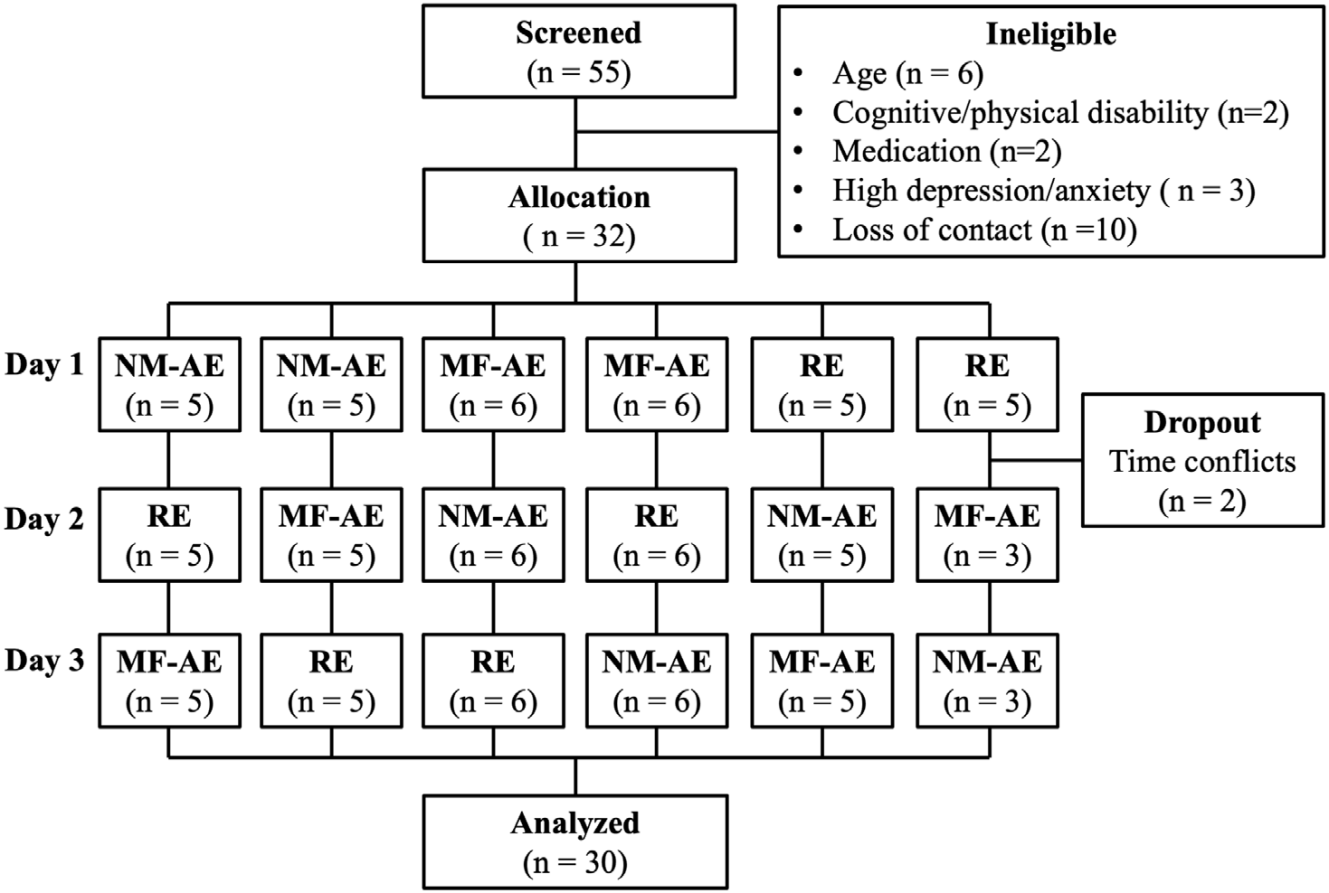
The CONSORT flow diagram of the experimental procedure.

During the first visit, participants completed the International Physical Activity Questionnaire Short Form (IPAQ‐SF) (Lee et al. 2011), five‐facet mindfulness questionnaire (FFMQ) (Baer et al. 2008), and emotion regulation questionnaire (ERQ) (Gross and John 2003) to assess physical activity (metabolic equivalent [MET] minutes per week), dispositional mindfulness, and habitual application of emotional regulation strategies: cognitive reappraisal (antecedent‐focused changing the interpretation of emotional events before a response occurs; internal consistency *α* = 0.83 and *ω* = 0.83) and expressive suppression (response‐focused inhibiting emotional expressions after a response has been activated; internal consistency *α* = 0.80 and *ω* = 0.81). A within‐subject crossover design was used for participants to complete MF‐AE, NM‐AE and rest conditions on three separate days that were scheduled at the same time of day and 7 days apart (difference in time of day: 36 ± 72 min; Days between visits: 7.5 ± 1.6 days) in counterbalanced order (Figure 2). Using a computer‐generated randomization scheme with 36 rows (e.g., six repetitions of each of the six possible intervention condition orders), participants were randomly allocated to one of the potential six intervention condition orders. This study procedure was not pre‐registered and both participants and experimenters were not blinded because of the nature of supervised interventions involving exercise and mindfulness. Participants were advised to wear appropriate exercise clothing and refrain from vigorous physical activity and stimulants such as coffee, tea, and alcohol at least 6 h before their visits. During each day before the intervention condition, participants provided FS and were fitted with an HR monitor and EEG Quik‐Cap. During each intervention, HR was assessed every 2 min. After the intervention, SMS‐PA2, FS, MES, PACES, RP, and PP were measured, followed by EEG recording during the picture‐viewing task.

### 2.8 Statistical Analysis

Analyses were performed using the *lme4*, *lmerTest*, and *emmeans* packages in RStudio (v 1.3.1093) with Kenward‐Roger degrees of freedom approximations. Multilevel modeling (MLM) was utilized to allow the inclusion of participants with missing data despite study completion.^1^ Analyses were conducted with *α* = 0.05. Follow‐up contrasts used to decompose significant main effects or interactions used the Bonferroni adjustment. Effect sizes were reported using partial eta‐squared (*η*_*p*_^2^) and Cohen's *d*, which was computed as the *M*_diff_ divided by the SD of the *M*_diff_.

To verify the manipulation of state‐mindfulness, scores from SMS‐PA2 dimensions and focuses were analyzed using a 3 (Condition: MF‐AE, NM‐AE, vs. rest) × 2 (Dimension: Monitoring vs. Accepting) × 2 (Focus: Body vs. Mind) univariate MLM. To verify the physical and mental exertion, mean exercise HR and MES were compared between three intervention conditions using a univariate MLM. Similar MLMs comparing conditions were conducted to analyze PACES, RP, and PP to determine the intervention effect on enjoyment and pleasure. FS was analyzed using a 3 (Condition: MF‐AE, NM‐AE, vs. rest) × 2 (Time: before vs. after) univariate MLM to determine the intervention effect on affective valence. Analysis on LPP amplitudes was conducted using a 3 (Condition: MF‐AE, NM‐AE, vs. rest) × 3 (Valence: pleasant, unpleasant, vs. neutral) univariate MLM. To test the primary hypothesis of the intervention effect on emotional reactivity, LPP_diff_ was analyzed using a 3 (Condition: MF‐AE, NM‐AE, vs. rest) × 2 (Reactivity: positive vs. negative) univariate MLM. Similar MLMs were used to perform the data‐driven post hoc analysis on LPP amplitudes and LPP_diff_ during the early (400–1200 ms) and later (1200–2000 ms) windows of stimulus presentation, respectively.

In addition to the fixed effects described in the models above, FFMQ was included as a covariate because it could contribute to the extent to which state‐related mindfulness is induced by interventions (Khatri et al. 2024). Consistent with suggestions on accounting for statistical dependencies in repeated measures factorial designs, participant and/or participant by within‐subjects main effect interactions were included as random intercepts when appropriate. For instance, the model to examine effects of Condition, Time, and their interactions on FS was specified as FS ~ Condition × Time + FFMQ + (1|Participant) + (1|Participant:Condition) + (1|Participant:Time), while the model examining effects of Condition, Valence, and their interactions on LPP amplitudes was specified as LPP ~ Condition × Valence + FFMQ + (1|Participant) + (1|Participant:Condition) + (1|Participant:Valence). These simplified models without including random slopes were used for parsimony and to avoid convergence issues. All models were estimated using restricted maximum likelihood.

## 3 Results

**FIGURE 3:**
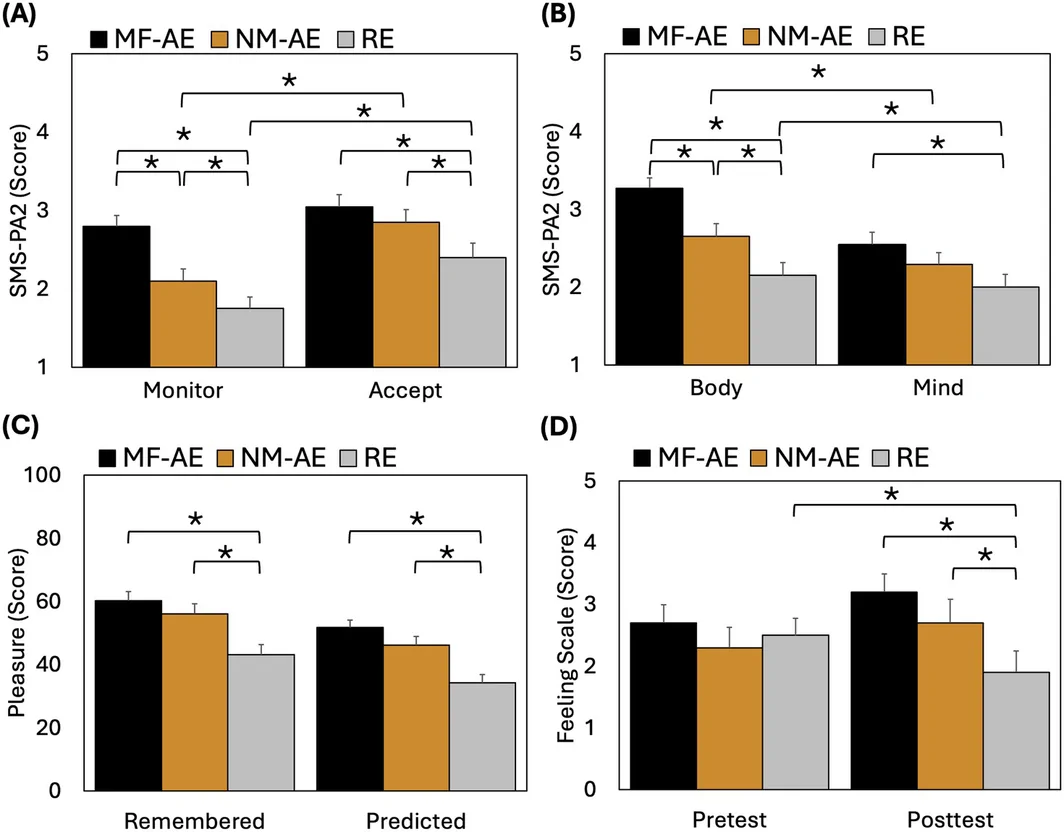
Difference in intervention target and affective measures between mindful aerobic exercise (MF‐AE), non‐mindful aerobic exercise (NM‐AE), and rest (RE) conditions. Sub‐plot (A) illustrates the Condition × Dimension interaction on SMS‐PA2 (State Mindfulness Scale for Physical Activity 2) scores. Sub‐plot (B) illustrates the Condition × Focus interaction on SMS‐PA2 scores. Sub‐plot (C) illustrates the Condition main effects on remembered pleasure and predicted pleasure, respectively. Sub‐plot (D) illustrates the Condition × Time interaction on the feeling scale. * = *p* < 0.05.

Participant characteristics and descriptives of outcome variables are shown in Tables 2, 3, 4. Analyses including Condition Order as an additional factor did not show any significant interaction involving Condition Order and Condition (*p*s > 0.05); therefore, condition order was not considered in any of the subsequent analyses. Statistically significant fixed effects involving the Condition effect are summarized in Table 5 (see supplement for the complete summary of all main and interaction effects). Significant main and interaction effects involving Condition on SMS‐PA2, pleasure, and FS were depicted in Figure 3.

**TABLE 2:** Descriptives of participant characteristics.

Variables	Mean ± standard deviation (range)
*N*	30 (15 male)
Age (years)	22.1 ± 2.2 (18–25)
Height (m)	1.67 ± 0.08 (1.53–1.82)
Weight (kg)	68.51 ± 13.54 (43.40–91.56)
BMI (kg/m^2^)	24.32 ± 3.20 (17.80–29.52)
IPAQ‐SF score	3582.19 ± 1411.22 (1013–6093)
DASS	
Depression	1.77 ± 1.87 (0–7)
Anxiety	1.93 ± 2.23 (0–7)
Stress	2.17 ± 2.77 (0–10)
FFMQ	
Observing_Avg_	3.44 ± 0.56 (2–5)
Describing_Avg_	3.35 ± 0.69 (2–5)
Acting with awareness_Avg_	3.13 ± 0.59 (2–4)
Non‐judging_Avg_	3.35 ± 0.86 (1–5)
Non‐reacting_Avg_	3.20 ± 0.55 (2–4)
Sum	16.47 ± 1.94 (13–21)
ERQ	
Cognitive reappraisal	32.50 ± 5.23 (17–42)
Expressive suppression	14.40 ± 4.80 (7–23)
Time between visits (day)	7.5 ± 1.6 (5.5–14)
Difference in time of visit (hour)	0.8 ± 1.4 (0–6.1)

Abbreviations: DASS, Depression, Anxiety, and Stress Scale; ERQ, Emotion Regulation Questionnaire; FFMQ, Five Facet Mindfulness Questionnaire; IPAQ‐SF, Physical Activity Questionnaire Short Form (metabolic equivalent minutes per week).

**TABLE 3:** Means and standard deviations of intervention target, affect, pleasure, and enjoyment under three experimental conditions.

Measure	MF‐AE	NM‐AE	Rest
HR	136 ± 1.7	135 ± 1.7	71 ± 9.8
SMS‐PA2			
Monitoring of body	3.3 ± 0.7	1.9 ± 0.9	1.8 ± 0.8
Monitoring of mind	2.3 ± 0.8	2.3 ± 0.8	1.7 ± 0.8
Accepting of body	3.3 ± 0.8	3 ± 0.9	2.5 ± 1
Accepting of mind	2.8 ± 0.9	2.7 ± 0.9	2.3 ± 1
MES	4 ± 1.9	4 ± 1.6	3 ± 2.1
FS pre	2.7 ± 1.6	2.3 ± 1.8	2.5 ± 1.5
FS post	3.2 ± 1.6	2.7 ± 2.1	1.9 ± 1.9
RP	60.2 ± 16.4	56.1 ± 17.4	43.2 ± 17.6
PP	51.8 ± 12.5	46.2 ± 15.2	34.3 ± 13.9
PACES	95 ± 18.0	90 ± 20.7	64 ± 17.7

Abbreviations: FS, Feeling Scale; HR, Heart Rate (beats per minute); MES, Mental Effort Scale; MF‐AE, Mindful aerobic exercise; NM‐AE, Non‐mindful aerobic exercise; PACES, Physical Activity Enjoyment Scale; PP, Predicted Pleasure; RE, Rest; RP, Remembered Pleasure; SMS‐PA 2, State Mindfulness Scale for Physical Activity 2.

**TABLE 4:** Means and standard deviations of emotional reactivity measures derived from different post‐stimulus windows following three experimental conditions.

Measure	MF‐AE	NM‐AE	Rest
Post‐stimulus 400–2000 ms window			
LPP pleasant	4.7 ± 4.2	4.0 ± 5.0	4.1 ± 4.7^a^
LPP unpleasant	4.7 ± 4.4	4.9 ± 5.5	5.8 ± 5.2^a^
LPP neutral	1.3 ± 3.6	1.1 ± 4.0	1.5 ± 3.9^a^
Positive LPP_diff_	3.3 ± 2.9	2.9 ± 2.7	2.5 ± 2.2^a^
Negative LPP_diff_	3.4 ± 3.0	3.8 ± 3.7	4.2 ± 2.5^a^
Post‐stimulus 400–1200 ms window			
LPP pleasant	5.2 ± 4.6	4.4 ± 5.1	4.7 ± 5.5^a^
LPP unpleasant	5.7 ± 4.7	5.8 ± 6.0	6.8 ± 6.0^a^
LPP neutral	1.9 ± 3.8	1.6 ± 4.3	2.1 ± 4.2^a^
Positive LPP_diff_	3.3 ± 3.0	2.8 ± 2.6	2.6 ± 2.6^a^
Negative LPP_diff_	3.8 ± 3.2	4.2 ± 4.1	4.7 ± 2.7^a^
Post‐stimulus 1200–2000 ms window			
LPP pleasant	4.2 ± 4.2	3.6 ± 5.1	3.5 ± 4.2^a^
LPP unpleasant	3.8 ± 4.3	4.0 ± 5.4	4.7 ± 4.6^a^
LPP neutral	0.7 ± 3.7	0.6 ± 4.0	1.0 ± 3.8^a^
Positive LPP_diff_	3.5 ± 3.1	3.0 ± 3.4	2.5 ± 1.9^a^
Negative LPP_diff_	3.0 ± 3.1	3.3 ± 4.1	3.8 ± 2.4^a^

Abbreviations: LPP, Late positive potential; LPP_diff_, LPP extracted from difference waves between valenced and neutral images; MF‐AE, Mindful aerobic exercise; NM‐AE, Non‐mindful aerobic exercise; RE, Rest.

^a^ Only 29 observations for LPP measures following rest condition due to technical error leading to one missing observation.

**TABLE 5:** Summary of statistically significant fixed effects from the univariate multilevel model analysis on intervention target, affect, pleasure, enjoyment, and emotional reactivity measures.

Measure	Effect	df	*F*	*p*	*η*_*p*_^2^
HR	Condition	2, 58	1274.3	< 0.001	0.98
SMS‐PA2	Condition	2, 58	30.0	< 0.001	0.51
	Dimension	1, 29	17.9	< 0.001	0.38
	Focus	1, 29	22.1	< 0.001	0.43
	Condition × Dimension	2, 203	4.8	0.009	0.05
	Condition × Focus	2, 203	5.4	0.005	0.05
MES	Condition	2, 58	3.7	0.030	0.11
FS	Condition	2, 58	5.3	0.008	0.15
	Condition × Time	2, 58	5.1	0.009	0.15
RP	Condition	2, 58	10.3	< 0.001	0.26
PP	Condition	2, 58	18.6	< 0.001	0.39
PACES	Condition	2, 58	34.8	< 0.001	0.55
400–2000 ms LPP	Valence	2, 58	59.2	< 0.001	0.67
400–2000 ms LPP_diff_	Reactivity	1, 29	9.8	0.004	0.25
	Condition × Reactivity	2, 57	4.3	0.011	0.13
400–1200 ms LPP	Valence	2, 58	57.8	< 0.001	0.67
400–1200 ms LPP_diff_	Reactivity	1, 29	23.3	< 0.001	0.45
	Condition × Reactivity	2, 57	4.0	0.023	0.12
1200–2000 ms LPP	Valence	2, 58	46.9	< 0.001	0.62
1200–2000 ms LPP_diff_	Condition × Reactivity	2, 57	3.8	0.029	0.12

Abbreviations: FS, Feeling Scale; HR, Heart Rate; LPP, Late positive potential; LPP_diff_, LPP extracted from difference waves between valenced and neutral images; MES, Mental Effort Scale; PACES, Physical Activity Enjoyment Scale; PP, Predicted Pleasure; RP, Remembered Pleasure; SMS‐PA 2, State Mindfulness Scale for Physical Activity 2.

### 3.1 State‐Related Mindfulness

There were main effects of Dimension and Focus, with scores being higher for accepting than the monitoring dimension and being higher for body than mind focus. The main Condition effect was also significant, with MF‐AE showing a higher overall score than NM‐AE (*t*[58] = 4.1, *p* < 0.001, *d* = 0.76) and rest (*t*[58] = 7.8, *p* < 0.001, *d* = 1.41) conditions and NM‐AE showing a higher overall score than rest condition (*t*[58] = 3.7, *p* = 0.001, *d* = 0.67). These effects were superseded by Condition × Dimension and Condition × Focus interactions. Decomposing the Condition × Dimension interaction examining Condition effect in each Dimension showed that MF‐AE and NM‐AE had higher scores than rest for both monitoring (MF‐AE: *t*[141] = 7.6, *p* < 0.001, *d* = 1.38; NM‐AE: *t*[141] = 2.6, *p* = 0.030, *d* = 0.48) and accepting (MF‐AE: *t*[141] = 4.5, *p* < 0.001, *d* = 0.81; NM‐AE: *t*[141] = 3.1, *p* = 0.008, *d* = 0.56) dimensions and that MF‐AE had a higher score than NM‐AE in monitoring dimension (*t*[141] = 5.0, *p* < 0.001, *d* = 0.91) but not accepting (*t*[141] = 1.4, *p* = 0.493, *d* = 0.26) dimension. Additionally, lower scores were found for the monitoring than accepting dimension following NM‐AE (*t*[71] = 4.4, *p* < 0.001, *d* = 0.81) and rest (*t*[71] = 4.1, *p* = 0.001, *d* = 0.74) conditions but no such dimension‐related difference was found following MF‐AE (*t*[71] = 1.4, *p* = 0.150, *d* = 0.26). Decomposing the Condition × Focus interaction examining condition‐related difference in each focus showed that MF‐AE (*t*[141] = 8.1, *p* < 0.001, *d* = 1.47) and NM‐AE (*t*[141] = 3.6, *p* = 0.002, *d* = 0.65) had higher scores than rest and that MF‐AE had a higher score than NM‐AE (*t*[141] = 4.5, *p* < 0.001, *d* = 0.82) in body focus. For the mind focus, MF‐AE had a higher score than rest (*t*[141] = 4.0, *p* = 0.003, *d* = 0.73) but NM‐AE did not differ from MF‐AE (*t*[141] = 1.9, *p* = 0.188, *d* = 0.34) or rest (*t*[141] = 2.1, *p* = 0.113, *d* = 0.38). Additionally, higher scores were found for the body than mind focus following MF‐AE (*t*[126] = 5.4, *p* < 0.001, *d* = 0.98) and NM‐AE (*t*[126] = 2.7, *p* = 0.008, *d* = 0.49) conditions but no such focus‐related difference was found following rest (*t*[126] = 1.1, *p* = 0.255, *d* = 0.21).

### 3.2 Physical and Mental Exertion

There were main effects of Condition on MES and HR. Post hoc analyses showed that MF‐AE and NM‐AE conditions resulted in higher HR (MF‐AE: *t*[58] = 43.9, *p* < 0.001, *d* = 8.02; NM‐AE: *t*[58] = 43.5, *p* < 0.001, *d* = 7.95) than the rest condition. No significant difference in HR was found between the MF‐AE and NM‐AE conditions (*t*[58] = 0.38, *p* > 0.999, *d* < 0.07). MES was higher following MF‐AE compared with rest (*t*[58] = 2.5, *p* = 0.047, *d* = 0.45) while there was no difference between NM‐AE and MF‐AE (*t*[58] = 0.3, *p* > 0.999, *d* = 0.05) or between NM‐AE and rest (*t*[58] = 2.2, *p* = 0.094, *d* = 0.40).

### 3.3 Affective Valence

There was a main effect of Condition that was superseded by the Condition × Time interaction. Decomposition of the Condition × Time interaction by examining the Condition effect at each time point indicated no differences at pretest (*t*s[111] < 1.3, *p*s > 0.612, *d*s < 0.23). However, at posttest, MF‐AE (*t*[111] = 4.4, *p* < 0.001, *d* = 0.80) and NM‐AE (*t*[111] = 2.6, *p* = 0.036, *d* = 0.47) conditions had more positive FS responses than rest. There was no difference in FS responses between MF‐AE and NM‐AE (*t*[111] = 1.8, *p* = 0.219, *d* = 0.33). When examining pre‐to‐posttest differences, there was a decrease in FS during the rest condition (*t*[81] = 2.0, *p* = 0.045, *d* = 0.37), while there were nonsignificant, positive changes in the MF‐AE (*t*[81] = 1.7, *p* = 0.091, *d* = 0.31) and NM‐AE (*t*[81] = 1.2, *p* = 0.242, *d* = 0.21) conditions.

### 3.4 Enjoyment and Pleasure

There were main effects of Condition on PACES, RP, and PP, with MF‐AE and NM‐AE conditions resulting in greater enjoyment and pleasure, as indexed by higher PACES (MF‐AE: *t*[58] = 7.8, *p* < 0.001, *d* = 1.42; NM‐AE: *t*[58] = 6.9, *p* < 0.001, *d* = 1.18), RP (MF‐AE: *t*[58] = 4.3, *p* < 0.001, *d* = 0.79; NM‐AE: *t*[58] = 3.3, *p* = 0.005, *d* = 0.60), and PP (MF‐AE: *t*[58] = 6.0, *p* < 0.001, *d* = 1.08; NM‐AE: *t*[58] = 4.1, *p* < 0.001, *d* = 0.74) scores than the rest condition. No significant difference in these measures was found between the MF‐AE and NM‐AE conditions (*t*s[58] < 1.9, *p*s > 0.183, *d*s < 0.35), despite PACES, RP, and PP scores being numerically higher for MF‐AE than NM‐AE.

### 3.5 Emotional Reactivity

Analysis of LPP amplitude showed a main effect of Valence, confirming image‐related LPP modulation by emotional content, with post hoc tests indicating that LPP amplitude was larger for pleasant (*t*[58] = 8.1, *p* < 0.001, *d* = 1.47) and unpleasant (*t*[58] = 10.4, *p* < 0.001, *d* = 1.89) compared with neutral images. LPP amplitude was also numerically larger for unpleasant relative to pleasant images, but this difference did not reach conventional statistical significance (*t*[58] = 2.3, *p* = 0.076, *d* = 0.42). No significant effect involving Condition was found.

Analysis of LPP_diff_ showed a main effect of Reactivity, with negative emotional reactivity being greater compared with positive emotional reactivity. This effect was superseded by a Condition × Reactivity interaction, indicating that although there was no between‐condition difference for either positive or negative LPP_diff_ (*t*s[88] < 1.4, *p*s > 0.455, *d*s < 0.26), negative LPP_diff_ was larger than positive LPP_diff_ following the NM‐AE (*t* = 2.1, *p* = 0.042, *d* = 0.38) and rest (*t* = 3.9, *p* < 0.001, *d* = 0.71) conditions but not following the MF‐AE condition (*t* = 0.1, *p* = 0.957, *d* = 0.01).

### 3.6 Emotional Reactivity by Early and Late Post‐Stimulus Time Window

Similar to the analysis using the entire picture‐viewing period, post hoc data‐driven analysis showed no significant effect involving Condition but a main effect of Valence for LPP amplitudes extracted from the 400–1200 ms and the 1200–2000 ms post‐stimulus windows. Across windows, pleasant (*ts*[58] > 7.3, *ps* < 0.001, *ds* > 1.32) and unpleasant (*ts*[58] > 8.8, *ps* < 0.001, *ds* > 1.61) images elicited larger LPP amplitudes compared with neutral images; however, LPP amplitude was larger for unpleasant than pleasant images only during the 400–1200 ms window (*t*[58] = 3.2, *p* = 0.005, *d* = 0.32) but not the 1200–2000 ms window.

There was a main Reactivity effect on LPP_diff_ during the 400–1200 ms and the 1200–2000 ms post‐stimulus windows, with both windows showing greater negative LPP_diff_ compared with positive LPP_diff_; however, this effect was superseded by a Condition × Reactivity interaction for LPP_diff_ derived from both windows. Although decomposition of this interaction on LPP_diff_ did not show any between‐condition difference across post‐stimulus windows (*t*s[84–89] < 1.6, *p*s > 0.319, *d*s < 0.30), there were unique condition‐specific reactivity effects on LPP_diff_ during these two post‐stimulus windows. In the 400–1200 ms window, negative LPP_diff_ was larger than positive LPP_diff_ following the NM‐AE (*t*[84] = 3.3, *p* = 0.001, *d* = 0.61) and rest (*t*[84] = 4.8, *p* < 0.001, *d* = 0.88) conditions but not following the MF‐AE condition (*t*[84] = 1.1, *p* = 0.272, *d* = 0.20). In the 1200–2000 ms window, negative LPP_diff_ was larger than positive LPP_diff_ following the rest (*t*[82] = 4.8, *p* < 0.001, *d* = 0.88) condition, while such a reactivity‐related modulation was nonsignificant following both the NM‐AE (*t*[81] = 0.7, *p* = 0.487, *d* = 0.13) and MF‐AE conditions (*t*[81] = 0.9, *p* = 0.368, *d* = 0.17).

## 4 Discussion

This study investigated the effects of a single 30 min session of MF‐AE on self‐reported affective responses and neural emotional reactivity outcomes compared with an active NM‐AE control and a passive rest control. While both MF‐AE and NM‐AE successfully increased HR and induced state‐related MF, MF‐AE resulted in high perceived cognitive effort and was effective for greater induction of MF in the monitoring dimension and across the body and mind focuses. Although more positive affective (FS, PACES, RP, and PP) outcomes were reported following MF‐AE and NM‐AE compared with the rest condition, they did not differ between the two exercise conditions. LPP amplitudes were larger in response to emotionally salient images, with unpleasant images eliciting larger LPP than pleasant images during the early stage (400–1200 ms) of emotional processing. Such a stronger LPP response to unpleasant images was supported by the LPP_diff_ analysis showing greater negative LPP_diff_ compared with positive LPP_diff_. Although the lack of between‐condition difference in LPP and LPP_diff_ failed to support the hypothesized combined effect of MF to AE on emotional reactivity, the stronger tendency in reacting to adverse stimuli emerged following the NM‐AE (*d* = 0.38) and rest (*d* = 0.71) conditions but was mitigated following the MF‐AE condition (*d* = 0.01). Post hoc analysis indicated a similar mitigation effect emerging only during the 1200–2000 ms post‐stimulus window following the NM‐AE condition, suggesting a temporally additive effect of MF and AE. Taken together, incorporating MF into AE can be an effective approach to not only improve affective outcomes associated with AE but also achieve a greater and more comprehensive state‐related MF while attenuating the early tendency to react to negative emotion‐eliciting stimuli.

The design in the MF‐AE intervention was effective for inducing state‐mindfulness without compromising the intended exercise intensity. Results from HR confirmed a similar moderate intensity achieved during both the MF‐AE and NM‐AE conditions. Although both MF‐AE and NM‐AE conditions resulted in increased state‐mindfulness in body focus than the rest control, MF‐AE had additional increases in body focus than NM‐AE and additional increases in mind focus than the rest control, likely due to a greater mental effort. These findings are consistent with previous research incorporating MF into 10 min moderate‐to‐vigorous intensity (Cox et al. 2018) and 20 min self‐paced (Cox et al. 2020) exercises. Through using the SMS‐PA2 that more precisely captured the monitoring and accepting dimensions of state‐mindfulness, results from the current study extended the previously reported higher overall state‐mindfulness induction following mindful AE (Ullrich‐French et al. 2024) by showing that integrating MF into AE could induce additional increases in the monitoring dimension beyond AE alone. This dimension‐specific increase likely stemmed from the relatively longer intervention duration with more frequent cues for awareness of body sensations, allowing greater induction of monitoring‐related state‐mindfulness.

Although both MF‐AE and NM‐AE led to more positive affect and greater enjoyment and pleasure, as indexed by higher FS, PACES, RP, and PP scores than the rest condition, no significant difference was observed between MF‐AE and NM‐AE conditions, suggesting that combining MF with AE did not influence affective valence, enjoyment, and pleasure. These findings were generally consistent with the positive acute exercise effects on affective outcomes (Reed and Ones 2006; Ekkekakis and Petruzzello 1999) but added the discrepancy to the literature regarding the role of MF to augment AE‐induced affective benefits (Cox et al. 2020, 2018; Ullrich‐French et al. 2024). For instance, Cox and colleagues found more positive affect and greater enjoyment following a 10 min mindful moderate‐to‐vigorous intensity AE than AE alone in one study (Cox et al. 2018) but the other studies found only more positive PP following a 20 min self‐paced AE with versus without MF (Cox et al. 2020) or no difference in these measures between 20 min mindful AE and AE alone at moderate‐to‐vigorous intensity (Ullrich‐French et al. 2024). However, these mixed results were based on studies including only female (Cox et al. 2020; Ullrich‐French et al. 2024) or predominantly female (> 82%) (Cox et al. 2018) and less physically active (e.g., < 150 min exercise per week) participants in a wider age range (18–58 years). Additionally, these studies were different in their uses in multiple assessments for affective valence during (i.e., every 4–5 min) (Cox et al. 2020, 2018; Ullrich‐French et al. 2024) and/or following exercise (i.e., immediately and delay after exercise) (Cox et al. 2018) compared with the current study including the evaluation only before and after exercise. The inclusion of only pretest and posttest without in‐task assessment in the current study was designed to avoid eliciting the monitoring and judgmental processes that could influence state‐mindfulness induction. However, this design in controlling for internal validity (e.g., state‐mindfulness induced by MF cues) may also fail to provide a robust estimate of exercise‐related changes in affective valence to best capture the augmenting effect of MF on AE‐related affect responses (Cox et al. 2018). Clearly, more research utilizing standardized approach to systematically manipulate exercise parameters and evaluate responses during different stages temporally relative to exercise onset/offset while considering individual difference in sex and physical activity levels is needed to better understand the role of MF to the affective effect of AE.

Beyond the self‐reported spontaneous affective outcomes, task‐induced emotional reactivity and its changes following MF‐AE relative to NM‐AE and rest were investigated to determine the potential combined effect of MF and AE on emotional reactivity. Consistent with earlier emotional reactivity studies, pleasant and unpleasant images elicited larger LPP amplitudes than neutral ones across intervention conditions, suggesting increases in motivated attention in response to emotionally laden stimuli (Hajcak and Foti 2020). Further, a larger LPP amplitude was observed in response to unpleasant compared with pleasant images and this difference reached statistical significance during the 400–1200 ms post‐stimulus, likely due to higher arousal ratings for unpleasant than pleasant images and negativity bias in facilitating the early processing of and response to aversive stimuli (Weinberg and Hajcak 2010). Such a stronger tendency in reacting to negatively emotion‐eliciting stimuli was also supported by the analysis on LPP_diff_, with negative reactivity (unpleasant–neutral) being larger than positive reactivity (pleasant–neutral) throughout the entire viewing process during the presentation of emotion‐eliciting images.

Despite the successful manipulation of valence‐related modulations in emotional reactivity measures, they did not differ across intervention conditions. This result neither supported a combined effect of AE and MF nor replicated the previously reported respective effects of these interventions on positive (Brush et al. 2020, 2021; Ligeza et al. 2023) and negative emotional reactivity (Lin et al. 2016, 2024; Uusberg et al. 2016). The absence of differences in unpleasant LPP or negative LPP_diff_ between the MF‐AE and NM‐AE conditions suggests that the MF induction procedure employed during AE may not be directly comparable to MF protocols delivered without physical exertion in prior studies (Lin et al. 2016, 2024).

In contrast to traditional protocols involving primarily sedentary MF practices (Lin et al. 2016, 2024), the current MF induction cues were specifically designed to synchronize monitoring and acceptance processes with AE. Although these exercise‐specific MF cues effectively increased state‐related mindfulness, this mindfulness induction did not translate into subsequent reduction in negative emotional reactivity measures. Similarly, the lack of AE‐related effects on positive LPP/LPP_diff_, regardless of the inclusion of MF cues, compared with the rest condition may result from the additional attentional demand for participants to listen to auditory stimuli while exercising. Unlike aversive stimuli that are processed quickly with less conscious monitoring, pleasant stimuli require more deliberate and conscious allocation of attention (Carretié et al. 2004, 2001). It is plausible that attending to MF cues and a health‐related podcast could have distracted and/or depleted the cognitive resources for deliberate/conscious attentional processes, leading to less resources available during the post‐intervention emotional engagement, particularly the intensity of the emotional reactions to images carrying positive valence and requiring conscious attention. This possibility may be especially relevant for the NM‐AE condition because the content of a health‐related podcast was asynchronized to the thought processes, breathing, and sensations of different body parts during exercise, creating a disadvantage for the NM‐AE condition to replicate the previously reported increases in positive LPP/LPP_diff_.

Alternatively, a few methodological differences between the current and previous studies, such as experimental design, picture‐viewing paradigm, and exercise prescription may also contribute to the observed null effect of NM‐AE on emotional reactivity. First, LPP was measured only following intervention in the current study, but previous studies included both pretest and posttest (Brush et al. 2021; Ligeza et al. 2023), allowing the control for individual difference in pre‐intervention positive LPP. However, the current study used a randomized counterbalanced approach and had participants complete each condition at a similar time of day; therefore, individual variability at pretest between conditions should be minimized. Second, most previous studies included two, instead of three, categories of images in the LPP‐eliciting tasks (Brush et al. 2021; Ligeza et al. 2023; Lin et al. 2016, 2024). For example, some studies included only neutral and pleasant images and only affiliative content and erotic images in the pleasant category in the picture‐viewing tasks to reduce the variability in positive emotional reactivity, maximally elicit positive emotional reactivity, and potentially improve statistical power to detect exercise‐induced effect on positive emotional reactivity (Brush et al. 2021). Some other work only included pleasant and unpleasant images to limit variability introduced by low‐arousal neutral images (Ligeza et al. 2023). The current study, instead, included pleasant, unpleasant, and neutral image types to allow more precise quantification of emotional reactivity and determination of intervention effects on different emotional reactivity domains. However, this approach may result in greater variability in LPP fluctuations due to the unpredictable sequence of image types and potential temporal interactions between image types, preventing the acute AE effect on positive emotional reactivity from showing. Third, the current study prescribed an individualized exercise intensity at around 70% of HR_max_ (HR = 135 ± 1.7), while several previous studies reported positive emotional reactivity changes following self‐paced AE corresponding to low‐to‐moderate intensity (HR = 121.7 ± 13.9 and 111.1 ± 18.0, respectively) (Brush et al. 2020, 2021). Given that more positive affective responses have been linked with exercise at a self‐selected intensity (Parfitt et al. 2006; Rose and Parfitt 2007), it is possible that the prescribed nature of the current NM‐AE protocol blunted the positive emotional reactivity effect of exercise.

Interestingly, within‐condition analyses revealed differential patterns of domain‐related differences in emotional reactivity across intervention conditions. In the analysis of LPP across the entire 2000 ms picture‐viewing period, the negative LPP_diff_ was significantly larger than the positive LPP_diff_ following the rest and NM‐AE conditions, while there was no reactivity‐related LPP_diff_ difference following the MF‐AE condition. Subsequent post hoc analyses indicated that this mitigation effect did not emerge during the earlier (400–1200 ms) post‐stimulus window but became evident during the later (1200–2000 ms) post‐stimulus window following the NM‐AE condition. The implications of these findings are two‐fold. First, AE may buffer against an initial negative reactivity tendency that persists throughout the entire image‐viewing period. This is not entirely unexpected as previous research reported an AE‐induced shift in attention away from negative emotional stimuli (Tian and Smith 2011; Rongrong and Jian 2024). Second, integrating MF cues simultaneously into AE may accelerate the onset of this mitigation effect. During the MF‐AE condition, participants were instructed to focus on their thoughts, breathing, and sensations throughout different areas of body with a nonjudging and accepting attitude, resulting in an associative mental engagement in exercise. It is possible that through active engagement of non‐evaluative monitoring of body and mind, participants' tendency toward negative relative to positive stimuli was reduced and this change was subsequently manifested by the early elimination of the difference between negative and positive LPP_diff_ and the sustained attenuation of this tendency through the picture‐viewing. This speculation was supported by the acute effect of MF induction on not only alleviating negative affectivity (e.g., depression, anxiety, stress) (Schumer et al. 2018) but also LPP‐measured emotional reactivity (Lin et al. 2016, 2024). Specifically, a brief MF induction focusing on open monitoring attenuated negative emotional reactivity, as evidenced by reduced LPP amplitudes in response to more negative arousing stimuli than neutral stimuli during a passive picture‐viewing paradigm with a prolonged viewing time (~5000 ms) (Lin et al. 2016, 2024). Taken together, incorporating a guided MF practice into AE may suppress the preferential processing for aversive stimuli, reducing the natural inclination toward stronger emotional reactions to negativity.

Several limitations of the study are worth noting. First, to maintain consistency in the stimuli‐activity mapping during each intervention condition, the podcasts used during the NM‐AE and rest conditions were not counterbalanced. That is, participants always listened to one podcast during the NM‐AE and the other one during the rest condition. This approach may have introduced potential bias (e.g., participants like one podcast more than the other) and confounded the influence of the NM‐AE and rest conditions on emotional reactivity, making the comparison between these conditions less comparable with previous studies contrasting AE and rest conditions without listening to podcasts (Brush et al. 2020, 2021). Second, a MF‐only condition was not included to quantify the MF effect on emotional reactivity. Future research including this additional active control is needed to more precisely determine whether the combined effect of MF and AE is synergistic or additive. Third, because LPP was only measured at the posttest, the potential influence of day‐to‐day variance on the differential patterns of LPP modulation following interventions cannot be eliminated. Fourth, given that the unpleasant images were also rated as higher in arousal, the observed mitigating effect on the difference between positive and negative reactivity following interventions may be interpreted as an attenuated tendency to react to stimuli including motivationally significant content (Hajcak and Foti 2020). Fifth, even though the current results indicate that the MF‐AE condition induced significantly higher state‐mindfulness compared with NM‐AE and rest conditions, it would be beneficial to further explore where participants allocated their attentional focus, whether being task‐related or unrelated, or internal or external (Cox et al. 2020; Ullrich‐French et al. 2024), to better understand how state‐mindfulness can be achieved. Lastly, the findings reported herein were based on an active sample (on average > 3000 MET per week) with a narrow age range. Future research including different age groups and activity levels is needed to improve generalizability.

In conclusion, while a single 30 min session of AE with and without MF both induced state‐mindfulness and similar levels of affect, pleasure, and enjoyment, integrating MF into AE via guided attentional instructions induced additional increases in state‐mindfulness in the monitoring dimension and across the body and mind focuses. Despite a high perceived mental effort associated with the engagement in MF cues during AE, this mind–body approach resulted in an earlier onset of emotional reactivity stability by reducing the tendency toward negatively valenced emotion‐eliciting events. These findings are significant in providing the preliminary evidence for the potential efficacy of simultaneously performing MF during AE in enhancing neurocognitive mechanisms underlying emotional processing and informing future refinement of therapeutic strategy for improving mental health.

## Author Contributions

**Wojciech Kielbus:** data curation, investigation, writing – original draft. **Salim Onbasi:** writing – original draft, data curation, project administration, writing – review and editing, investigation, methodology, conceptualization, formal analysis, supervision. **Yu‐Kai Chang:** writing – review and editing, conceptualization, validation. **Sarah Ullrich‐French:** methodology, supervision, validation, writing – review and editing. **Steve Amireault:** supervision, writing – review and editing, funding acquisition. **Shih‐Chun Kao:** conceptualization, investigation, methodology, validation, software, formal analysis, data curation, supervision, resources, project administration, writing – original draft, funding acquisition, visualization, writing – review and editing. **C. J. Brush:** conceptualization, investigation, methodology, formal analysis, validation, writing – review and editing, supervision.

## Funding

This work was supported by the Purdue Center for Research on Brain, Behavior, and NeuroRehabilitation (CEREBBRAL).

## Conflicts of Interest

The authors declare no conflicts of interest.

## Supporting information


**Table S1:** The stimuli used in the passive‐viewing task.
**Table S2:** Valence ratings for each neutral stimulus as reported by each subject.
**Table S3:** Arousal ratings for each neutral stimulus as reported by each subject.
**Table S4:** Valence ratings for each pleasant stimulus as reported by each subject.
**Table S5:** Arousal ratings for each pleasant stimulus as reported by each subject.
**Table S6:** Valence ratings for each unpleasant stimulus as reported by each subject.
**Table S7:** Arousal ratings for each unpleasant stimulus as reported by each subject.
**Table S8:** Mean valence and arousal ratings of stimuli as reported by each subject.
**Table S9:** Complete summary of statistics from the univariate multilevel model analysis on intervention target, affect, pleasure, enjoyment, and emotional reactivity measures.

## Data Availability

The data that support the findings of this study are available on request from the corresponding author. The data are not publicly available due to privacy or ethical restrictions.

## Endnotes

^1^
One participant completed the entire study but had missing LPP data from the rest control condition due to computer errors. The two participants who withdrew from the study were not included for analysis because they only participated in Day 1 and did not complete all measures involved in Day 1 due to time conflict and loss of interest.
