# Supplementary material for: Altering Affective Responses and Emotional Reactivity Through Acute Bouts of Aerobic Exercise With and Without Mindfulness
Source: Psychophysiology. 2026 Sep 25;63(9):e70405. doi: 10.1111/psyp.70405 (PMC13614478; doi:10.1111/psyp.70405)
Supplement: Supplementary file 1 [file PSYP-63-e70405-s001.docx]

Supplement

**Table S1** *The stimuli used in the passive-viewing task.*

| **Picture Valence** | **IAPS Stimulus Number** |
| --- | --- |
| Neutral | 5390, 5471, 5500, 5510, 5532, 5534, 5740, 7000, 7002, 7004, 7006, 7009, 7010, 7020, 7025, 7030, 7034, 7035, 7040, 7041, 7055, 7060, 7061, 7080, 7081, 7090, 7096, 7100, 7130, 7140, 7150, 7161, 7175, 7217, 7224, 7233, 7234, 7235, 7491, 7500, 7546, 7547 |
| Pleasant | 1441,1463, 1540, 1601, 1710, 1722, 1731, 1811, 1920, 2040, 2070, 2071, 2080, 2091, 2150, 2158, 2165, 2209, 2224, 2340, 2345, 2346, 2550, 4532, 4572, 4597, 4604, 4607, 4608, 4610, 4611, 4612, 4623, 4624, 4625, 4640, 4641, 4643, 4645, 4650, 4653, 4700 |
| Unpleseant | 1050, 1110, 1113, 1200, 1300, 1301, 1930, 2095, 3010, 3015, 3016, 3030, 3051, 3101, 3102, 3103, 3110, 3120, 3140, 3168, 3190, 3261, 3266, 3400, 3530, 6210, 6240, 6250, 6312, 6313, 6315, 6520, 6540, 6550, 6560, 6571, 9400, 9405, 9419, 9430, 9920, 9927 |
| *Note*. *IAPS = International Affective Picture System*. | |

**Table S2** *Valence ratings for each neutral stimulus as reported by each subject.*

| **Stimuli Codes** | **Subjects** | | | | | | | | | | | | | | | | | | | | | | | | | | | | |
| --- | --- | --- | --- | --- | --- | --- | --- | --- | --- | --- | --- | --- | --- | --- | --- | --- | --- | --- | --- | --- | --- | --- | --- | --- | --- | --- | --- | --- | --- |
|  | **S**  **1** | **S**  **2** | **S**  **3** | **S**  **4** | **S**  **5** | **S**  **6** | **S**  **7** | **S**  **8** | **S**  **9** | **S**  **10** | **S**  **11** | **S**  **12** | **S**  **13** | **S**  **14** | **S**  **15** | **S**  **16** | **S**  **17** | **S**  **18** | **S**  **19** | **S**  **20** | **S**  **21** | **S**  **22** | **S**  **23** | **S**  **24** | **S**  **25** | **S**  **26** | **S**  **27** | **S**  **28** | **S**  **29** |
| **5390** | 5 | 7 | 5 | 6 | 5 | 5 | 5 | 9 | 5 | 5 | 5 | 5 | 7 | 5 | 6 | 5 | 6 | 5 | 5 | 5 | 7 | 8 | 7 | 5 | 6 | 5 | 5 | 5 | 6 |
| **5471** | 5 | 5 | 5 | 5 | 4 | 5 | 5 | 8 | 5 | 7 | 5 | 5 | 5 | 5 | 5 | 5 | 1 | 5 | 1 | 5 | 5 | 6 | 7 | 6 | 6 | 5 | 5 | 5 | 7 |
| **5500** | 5 | 3 | 5 | 5 | 1 | 5 | 5 | 8 | 5 | 5 | 5 | 5 | 5 | 5 | 5 | 5 | 5 | 5 | 5 | 5 | 5 | 5 | 5 | 3 | 7 | 5 | 6 | 2 | 5 |
| **5510** | 5 | 3 | 5 | 7 | 1 | 2 | 6 | 9 | 5 | 5 | 5 | 5 | 5 | 5 | 5 | 5 | 5 | 5 | 5 | 5 | 5 | 6 | 5 | 4 | 6 | 5 | 6 | 1 | 5 |
| **5532** | 5 | 3 | 5 | 5 | 5 | 5 | 5 | 7 | 5 | 5 | 5 | 5 | 6 | 5 | 5 | 5 | 4 | 5 | 5 | 4 | 5 | 3 | 5 | 5 | 6 | 6 | 5 | 5 | 3 |
| **5534** | 5 | 2 | 5 | 5 | 1 | 5 | 5 | 9 | 5 | 5 | 5 | 5 | 5 | 5 | 5 | 5 | 5 | 5 | 5 | 5 | 5 | 5 | 5 | 3 | 6 | 4 | 6 | 1 | 4 |
| **5740** | 6 | 5 | 5 | 6 | 1 | 5 | 5 | 7 | 5 | 7 | 6 | 5 | 5 | 6 | 5 | 5 | 5 | 6 | 5 | 5 | 5 | 5 | 5 | 5 | 7 | 5 | 5 | 5 | 5 |
| **7000** | 5 | 3 | 5 | 5 | 5 | 5 | 6 | 5 | 5 | 5 | 5 | 5 | 4 | 5 | 5 | 5 | 5 | 4 | 5 | 6 | 6 | 6 | 5 | 5 | 5 | 5 | 5 | 5 | 5 |
| **7002** | 5 | 2 | 5 | 5 | 1 | 5 | 6 | 6 | 5 | 5 | 5 | 5 | 5 | 5 | 5 | 5 | 5 | 5 | 5 | 5 | 5 | 5 | 5 | 2 | 5 | 5 | 5 | 5 | 5 |
| **7004** | 5 | 4 | 5 | 5 | 5 | 5 | 6 | 5 | 5 | 5 | 5 | 5 | 5 | 6 | 5 | 5 | 5 | 5 | 5 | 5 | 5 | 5 | 5 | 2 | 6 | 5 | 5 | 5 | 5 |
| **7006** | 5 | 4 | 5 | 5 | 5 | 5 | 6 | 5 | 5 | 5 | 5 | 5 | 5 | 5 | 5 | 5 | 5 | 4 | 5 | 5 | 5 | 5 | 5 | 5 | 5 | 5 | 5 | 5 | 5 |
| **7009** | 5 | 5 | 5 | 5 | 5 | 5 | 7 | 7 | 5 | 5 | 5 | 5 | 5 | 5 | 5 | 5 | 5 | 5 | 5 | 5 | 5 | 5 | 5 | 1 | 5 | 5 | 6 | 5 | 5 |
| **7010** | 5 | 3 | 5 | 5 | 5 | 5 | 5 | 9 | 5 | 5 | 5 | 5 | 4 | 5 | 5 | 5 | 5 | 5 | 5 | 4 | 5 | 5 | 5 | 4 | 5 | 5 | 5 | 5 | 6 |
| **7020** | 5 | 3 | 5 | 5 | 5 | 5 | 6 | 5 | 5 | 5 | 5 | 5 | 4 | 5 | 5 | 5 | 5 | 4 | 5 | 5 | 5 | 4 | 5 | 5 | 5 | 5 | 4 | 5 | 5 |
| **7025** | 5 | 5 | 5 | 5 | 5 | 5 | 5 | 5 | 5 | 5 | 5 | 5 | 4 | 5 | 5 | 5 | 5 | 4 | 5 | 5 | 5 | 5 | 5 | 2 | 5 | 5 | 5 | 5 | 5 |
| **7030** | 5 | 3 | 5 | 5 | 5 | 5 | 4 | 5 | 5 | 5 | 5 | 5 | 4 | 5 | 5 | 5 | 6 | 5 | 5 | 5 | 5 | 5 | 5 | 5 | 5 | 5 | 5 | 5 | 5 |
| **7034** | 5 | 3 | 5 | 3 | 5 | 5 | 5 | 5 | 5 | 5 | 6 | 5 | 5 | 5 | 5 | 4 | 4 | 4 | 5 | 4 | 6 | 5 | 5 | 3 | 4 | 5 | 4 | 5 | 5 |
| **7035** | 5 | 4 | 1 | 5 | 5 | 5 | 6 | 6 | 5 | 5 | 5 | 5 | 5 | 5 | 5 | 5 | 5 | 4 | 5 | 5 | 5 | 5 | 5 | 5 | 5 | 5 | 5 | 5 | 5 |
| **7040** | 5 | 3 | 5 | 5 | 5 | 5 | 5 | 7 | 5 | 5 | 5 | 5 | 4 | 5 | 5 | 5 | 5 | 4 | 5 | 5 | 5 | 4 | 5 | 5 | 5 | 5 | 4 | 5 | 5 |
| **7041** | 5 | 5 | 5 | 5 | 5 | 5 | 6 | 5 | 5 | 5 | 5 | 5 | 5 | 5 | 5 | 5 | 5 | 6 | 5 | 5 | 5 | 5 | 5 | 5 | 5 | 5 | 5 | 5 | 6 |
| **7055** | 5 | 3 | 5 | 5 | 5 | 5 | 5 | 5 | 5 | 5 | 5 | 5 | 5 | 5 | 5 | 5 | 6 | 5 | 5 | 5 | 5 | 5 | 5 | 5 | 5 | 5 | 5 | 5 | 5 |
| **7060** | 5 | 3 | 5 | 5 | 5 | 5 | 5 | 5 | 5 | 5 | 4 | 5 | 4 | 5 | 5 | 5 | 5 | 4 | 5 | 5 | 5 | 5 | 5 | 5 | 5 | 5 | 4 | 5 | 4 |
| **7061** | 5 | 5 | 5 | 6 | 5 | 7 | 7 | 6 | 5 | 5 | 6 | 5 | 6 | 5 | 5 | 6 | 7 | 5 | 5 | 5 | 5 | 7 | 6 | 5 | 5 | 6 | 4 | 5 | 5 |
| **7080** | 5 | 4 | 5 | 5 | 5 | 5 | 7 | 5 | 5 | 5 | 5 | 5 | 5 | 5 | 5 | 5 | 5 | 5 | 5 | 1 | 5 | 6 | 5 | 5 | 5 | 5 | 5 | 5 | 5 |
| **7081** | 5 | 5 | 6 | 5 | 5 | 5 | 5 | 5 | 5 | 5 | 6 | 5 | 7 | 5 | 5 | 5 | 6 | 5 | 5 | 4 | 5 | 6 | 5 | 5 | 5 | 5 | 5 | 5 | 5 |
| **7090** | 5 | 4 | 5 | 6 | 5 | 4 | 7 | 7 | 5 | 5 | 5 | 5 | 5 | 4 | 5 | 5 | 7 | 5 | 6 | 5 | 5 | 6 | 5 | 5 | 6 | 5 | 4 | 5 | 6 |
| **7096** | 5 | 5 | 5 | 5 | 5 | 5 | 5 | 5 | 5 | 5 | 5 | 5 | 5 | 5 | 5 | 5 | 5 | 6 | 5 | 5 | 5 | 5 | 5 | 5 | 6 | 5 | 5 | 5 | 5 |
| **7100** | 5 | 4 | 5 | 5 | 5 | 5 | 5 | 5 | 5 | 5 | 4 | 5 | 6 | 5 | 5 | 5 | 5 | 6 | 5 | 5 | 5 | 5 | 6 | 5 | 6 | 5 | 5 | 5 | 5 |
| **7130** | 5 | 5 | 5 | 5 | 5 | 5 | 5 | 5 | 5 | 5 | 5 | 5 | 6 | 5 | 5 | 5 | 5 | 6 | 5 | 4 | 5 | 5 | 5 | 1 | 5 | 5 | 5 | 5 | 5 |
| **7140** | 5 | 4 | 5 | 5 | 5 | 5 | 5 | 5 | 5 | 5 | 5 | 5 | 7 | 5 | 5 | 5 | 5 | 6 | 5 | 5 | 5 | 5 | 5 | 5 | 6 | 5 | 5 | 5 | 5 |
| **7150** | 5 | 4 | 5 | 5 | 5 | 5 | 6 | 5 | 5 | 5 | 5 | 5 | 5 | 5 | 5 | 5 | 5 | 6 | 5 | 5 | 5 | 5 | 5 | 5 | 6 | 5 | 5 | 5 | 5 |
| **7161** | 5 | 5 | 5 | 5 | 5 | 5 | 5 | 5 | 5 | 5 | 4 | 5 | 5 | 5 | 5 | 5 | 5 | 4 | 5 | 6 | 5 | 4 | 5 | 6 | 5 | 5 | 5 | 5 | 5 |
| **7175** | 5 | 4 | 5 | 5 | 5 | 5 | 5 | 5 | 5 | 5 | 5 | 5 | 5 | 5 | 5 | 5 | 5 | 5 | 5 | 4 | 5 | 7 | 5 | 5 | 5 | 5 | 5 | 5 | 5 |
| **7217** | 5 | 4 | 5 | 5 | 5 | 5 | 6 | 5 | 5 | 5 | 5 | 5 | 5 | 5 | 5 | 6 | 5 | 4 | 4 | 1 | 5 | 5 | 7 | 5 | 5 | 5 | 4 | 5 | 5 |
| **7224** | 5 | 5 | 5 | 5 | 5 | 5 | 4 | 5 | 5 | 5 | 4 | 5 | 4 | 5 | 5 | 5 | 5 | 3 | 5 | 5 | 5 | 5 | 5 | 5 | 5 | 5 | 4 | 5 | 5 |
| **7233** | 5 | 4 | 5 | 5 | 5 | 5 | 6 | 5 | 5 | 5 | 5 | 5 | 6 | 6 | 5 | 6 | 5 | 5 | 5 | 5 | 5 | 7 | 6 | 5 | 7 | 5 | 4 | 5 | 5 |
| **7234** | 5 | 5 | 5 | 5 | 5 | 4 | 5 | 5 | 5 | 5 | 5 | 5 | 4 | 5 | 5 | 5 | 5 | 4 | 5 | 4 | 5 | 5 | 5 | 5 | 5 | 5 | 5 | 5 | 5 |
| **7235** | 5 | 5 | 5 | 5 | 5 | 5 | 5 | 4 | 5 | 5 | 5 | 5 | 5 | 5 | 5 | 5 | 5 | 5 | 5 | 5 | 5 | 5 | 5 | 5 | 5 | 5 | 5 | 5 | 6 |
| **7491** | 5 | 3 | 5 | 5 | 5 | 5 | 2 | 4 | 5 | 5 | 4 | 5 | 5 | 5 | 4 | 5 | 4 | 4 | 5 | 5 | 5 | 5 | 5 | 5 | 5 | 5 | 4 | 5 | 4 |
| **7500** | 5 | 5 | 5 | 5 | 5 | 5 | 5 | 6 | 5 | 5 | 4 | 5 | 5 | 5 | 5 | 5 | 4 | 5 | 5 | 5 | 5 | 6 | 5 | 5 | 7 | 5 | 5 | 5 | 6 |
| **7546** | 5 | 4 | 5 | 6 | 5 | 5 | 6 | 7 | 5 | 5 | 6 | 5 | 6 | 5 | 5 | 6 | 6 | 5 | 5 | 6 | 6 | 7 | 5 | 5 | 6 | 6 | 5 | 5 | 5 |
| **7547** | 5 | 5 | 5 | 5 | 5 | 5 | 5 | 5 | 5 | 5 | 5 | 5 | 5 | 5 | 5 | 5 | 4 | 5 | 5 | 5 | 5 | 5 | 5 | 5 | 6 | 5 | 5 | 5 | 7 |
| *Note. The stimulus codes presented in this table were derived from the International Affective Picture System (IAPS). The ratings were collected using the Self-Assessment Manikin (SAM). The letter “S” denotes a subject.* | | | | | | | | | | | | | | | | | | | | | | | | | | | | | |

**Table S3** *Arousal ratings for each neutral stimulus as reported by each subject.*

| **Stimuli Codes** | **Subjects** | | | | | | | | | | | | | | | | | | | | | | | | | | | | |
| --- | --- | --- | --- | --- | --- | --- | --- | --- | --- | --- | --- | --- | --- | --- | --- | --- | --- | --- | --- | --- | --- | --- | --- | --- | --- | --- | --- | --- | --- |
|  | **S**  **1** | **S**  **2** | **S**  **3** | **S**  **4** | **S**  **5** | **S**  **6** | **S**  **7** | **S**  **8** | **S**  **9** | **S**  **10** | **S**  **11** | **S**  **12** | **S**  **13** | **S**  **14** | **S**  **15** | **S**  **16** | **S**  **17** | **S**  **18** | **S**  **19** | **S**  **20** | **S**  **21** | **S**  **22** | **S**  **23** | **S**  **24** | **S**  **25** | **S**  **26** | **S**  **27** | **S**  **28** | **S**  **29** |
| **5390** | 1 | 4 | 1 | 1 | 1 | 2 | 1 | 6 | 1 | 1 | 2 | 1 | 2 | 1 | 1 | 1 | 2 | 1 | 1 | 5 | 2 | 1 | 1 | 1 | 2 | 2 | 3 | 1 | 1 |
| **5471** | 5 | 1 | 5 | 2 | 1 | 2 | 1 | 5 | 1 | 1 | 1 | 1 | 1 | 1 | 2 | 1 | 2 | 1 | 1 | 3 | 5 | 1 | 2 | 1 | 2 | 1 | 3 | 5 | 5 |
| **5500** | 2 | 2 | 5 | 2 | 1 | 3 | 2 | 8 | 1 | 1 | 1 | 1 | 1 | 1 | 1 | 2 | 5 | 1 | 1 | 5 | 1 | 1 | 3 | 1 | 2 | 2 | 2 | 1 | 5 |
| **5510** | 2 | 7 | 5 | 1 | 1 | 2 | 3 | 9 | 1 | 1 | 2 | 1 | 1 | 1 | 2 | 2 | 1 | 1 | 1 | 5 | 1 | 1 | 1 | 1 | 2 | 2 | 3 | 1 | 5 |
| **5532** | 1 | 5 | 1 | 1 | 1 | 2 | 1 | 4 | 1 | 1 | 1 | 1 | 3 | 1 | 1 | 1 | 3 | 1 | 1 | 4 | 1 | 1 | 1 | 1 | 2 | 2 | 2 | 1 | 2 |
| **5534** | 2 | 3 | 5 | 1 | 1 | 2 | 1 | 2 | 1 | 1 | 1 | 1 | 1 | 1 | 1 | 2 | 1 | 1 | 1 | 4 | 1 | 1 | 1 | 1 | 2 | 6 | 3 | 1 | 2 |
| **5740** | 3 | 2 | 5 | 1 | 1 | 2 | 1 | 2 | 1 | 1 | 1 | 1 | 1 | 1 | 1 | 1 | 1 | 1 | 1 | 5 | 1 | 2 | 1 | 1 | 3 | 1 | 2 | 1 | 1 |
| **7000** | 1 | 4 | 3 | 1 | 5 | 2 | 2 | 5 | 1 | 1 | 1 | 1 | 1 | 1 | 1 | 1 | 1 | 1 | 1 | 1 | 1 | 1 | 1 | 1 | 1 | 1 | 1 | 1 | 1 |
| **7002** | 1 | 1 | 5 | 1 | 1 | 2 | 2 | 5 | 1 | 1 | 1 | 1 | 1 | 1 | 1 | 1 | 1 | 1 | 1 | 4 | 1 | 1 | 1 | 1 | 1 | 1 | 1 | 1 | 1 |
| **7004** | 1 | 1 | 5 | 1 | 1 | 2 | 2 | 5 | 2 | 1 | 1 | 1 | 1 | 2 | 1 | 1 | 1 | 1 | 1 | 1 | 1 | 1 | 1 | 1 | 2 | 1 | 3 | 1 | 1 |
| **7006** | 1 | 1 | 1 | 1 | 1 | 2 | 3 | 1 | 1 | 1 | 1 | 1 | 1 | 1 | 1 | 1 | 1 | 1 | 1 | 1 | 1 | 1 | 1 | 1 | 1 | 1 | 1 | 1 | 1 |
| **7009** | 1 | 1 | 5 | 1 | 1 | 2 | 3 | 5 | 1 | 1 | 1 | 1 | 1 | 1 | 1 | 1 | 1 | 1 | 1 | 1 | 1 | 2 | 1 | 2 | 1 | 1 | 2 | 1 | 1 |
| **7010** | 1 | 2 | 5 | 1 | 1 | 2 | 1 | 5 | 1 | 1 | 1 | 1 | 2 | 1 | 1 | 1 | 1 | 1 | 1 | 1 | 1 | 1 | 1 | 1 | 2 | 1 | 2 | 1 | 1 |
| **7020** | 1 | 4 | 1 | 1 | 1 | 2 | 3 | 2 | 1 | 1 | 1 | 1 | 2 | 1 | 1 | 1 | 1 | 1 | 1 | 1 | 1 | 1 | 1 | 1 | 1 | 1 | 1 | 1 | 1 |
| **7025** | 1 | 1 | 5 | 1 | 1 | 3 | 1 | 5 | 1 | 1 | 2 | 1 | 2 | 1 | 1 | 1 | 1 | 1 | 1 | 5 | 1 | 1 | 1 | 1 | 1 | 1 | 1 | 1 | 1 |
| **7030** | 1 | 2 | 1 | 1 | 1 | 2 | 5 | 2 | 1 | 1 | 1 | 1 | 3 | 1 | 1 | 1 | 1 | 1 | 1 | 1 | 1 | 1 | 1 | 1 | 1 | 1 | 1 | 1 | 2 |
| **7034** | 3 | 3 | 5 | 2 | 1 | 2 | 1 | 5 | 1 | 1 | 7 | 1 | 1 | 1 | 1 | 2 | 5 | 2 | 1 | 5 | 1 | 2 | 2 | 1 | 2 | 1 | 2 | 1 | 1 |
| **7035** | 2 | 2 | 1 | 1 | 1 | 2 | 2 | 5 | 1 | 1 | 1 | 1 | 1 | 1 | 1 | 1 | 1 | 1 | 1 | 1 | 1 | 1 | 1 | 1 | 2 | 1 | 2 | 1 | 1 |
| **7040** | 1 | 1 | 1 | 1 | 1 | 2 | 2 | 5 | 1 | 1 | 1 | 1 | 2 | 1 | 1 | 1 | 1 | 1 | 1 | 1 | 1 | 1 | 1 | 1 | 1 | 1 | 2 | 1 | 1 |
| **7041** | 1 | 3 | 1 | 1 | 1 | 2 | 3 | 2 | 1 | 1 | 1 | 1 | 1 | 1 | 1 | 1 | 1 | 1 | 1 | 3 | 1 | 1 | 1 | 1 | 1 | 1 | 1 | 1 | 1 |
| **7055** | 2 | 1 | 1 | 1 | 1 | 2 | 1 | 5 | 1 | 1 | 1 | 1 | 1 | 1 | 1 | 1 | 2 | 1 | 1 | 1 | 1 | 1 | 1 | 1 | 2 | 1 | 2 | 1 | 1 |
| **7060** | 1 | 3 | 1 | 1 | 1 | 2 | 1 | 5 | 1 | 1 | 1 | 1 | 1 | 1 | 1 | 1 | 1 | 1 | 1 | 1 | 1 | 1 | 1 | 1 | 1 | 1 | 2 | 1 | 2 |
| **7061** | 1 | 3 | 1 | 2 | 1 | 6 | 2 | 5 | 1 | 1 | 3 | 1 | 3 | 1 | 1 | 2 | 3 | 1 | 1 | 1 | 1 | 2 | 1 | 1 | 2 | 5 | 1 | 1 | 1 |
| **7080** | 1 | 2 | 1 | 1 | 1 | 3 | 3 | 2 | 1 | 1 | 1 | 1 | 1 | 1 | 1 | 1 | 1 | 1 | 1 | 1 | 1 | 1 | 1 | 1 | 2 | 1 | 2 | 1 | 1 |
| **7081** | 1 | 2 | 4 | 1 | 1 | 3 | 1 | 4 | 1 | 1 | 3 | 1 | 2 | 1 | 1 | 1 | 4 | 2 | 1 | 3 | 1 | 1 | 2 | 1 | 2 | 1 | 2 | 1 | 1 |
| **7090** | 1 | 2 | 1 | 1 | 1 | 2 | 3 | 2 | 1 | 1 | 1 | 1 | 1 | 2 | 1 | 1 | 3 | 1 | 1 | 4 | 1 | 1 | 1 | 1 | 2 | 1 | 2 | 1 | 2 |
| **7096** | 1 | 1 | 1 | 1 | 1 | 2 | 1 | 2 | 1 | 1 | 2 | 1 | 1 | 1 | 1 | 2 | 1 | 3 | 1 | 1 | 1 | 1 | 1 | 2 | 2 | 1 | 1 | 1 | 1 |
| **7100** | 1 | 1 | 1 | 1 | 1 | 2 | 1 | 1 | 1 | 1 | 1 | 1 | 1 | 1 | 1 | 2 | 1 | 2 | 1 | 1 | 1 | 1 | 1 | 2 | 2 | 1 | 1 | 1 | 1 |
| **7130** | 1 | 3 | 1 | 1 | 1 | 2 | 1 | 2 | 1 | 1 | 2 | 1 | 2 | 1 | 1 | 1 | 1 | 2 | 1 | 1 | 1 | 2 | 2 | 1 | 2 | 1 | 2 | 1 | 1 |
| **7140** | 1 | 2 | 1 | 1 | 1 | 2 | 1 | 2 | 1 | 1 | 2 | 1 | 2 | 1 | 1 | 1 | 1 | 2 | 1 | 1 | 1 | 1 | 2 | 1 | 2 | 1 | 2 | 1 | 1 |
| **7150** | 1 | 1 | 1 | 1 | 1 | 2 | 2 | 2 | 1 | 1 | 1 | 1 | 1 | 1 | 1 | 1 | 1 | 1 | 1 | 1 | 1 | 1 | 2 | 1 | 2 | 1 | 1 | 1 | 1 |
| **7161** | 1 | 1 | 1 | 1 | 1 | 2 | 1 | 3 | 1 | 1 | 1 | 1 | 1 | 1 | 1 | 1 | 1 | 2 | 1 | 4 | 1 | 2 | 1 | 2 | 1 | 1 | 2 | 1 | 1 |
| **7175** | 1 | 2 | 1 | 1 | 1 | 2 | 1 | 1 | 1 | 1 | 1 | 1 | 1 | 1 | 1 | 1 | 1 | 1 | 1 | 5 | 1 | 2 | 1 | 1 | 2 | 1 | 2 | 1 | 1 |
| **7217** | 1 | 2 | 1 | 1 | 1 | 2 | 1 | 1 | 1 | 1 | 1 | 1 | 1 | 1 | 1 | 2 | 1 | 1 | 1 | 1 | 1 | 1 | 1 | 1 | 1 | 1 | 2 | 1 | 1 |
| **7224** | 1 | 2 | 1 | 1 | 1 | 2 | 2 | 3 | 1 | 1 | 1 | 1 | 4 | 1 | 1 | 1 | 1 | 1 | 1 | 1 | 1 | 1 | 1 | 1 | 1 | 1 | 2 | 1 | 1 |
| **7233** | 1 | 2 | 1 | 1 | 1 | 2 | 4 | 2 | 1 | 1 | 1 | 1 | 3 | 1 | 1 | 2 | 1 | 1 | 1 | 1 | 1 | 1 | 1 | 1 | 2 | 1 | 2 | 1 | 1 |
| **7234** | 1 | 2 | 1 | 1 | 1 | 2 | 1 | 1 | 1 | 1 | 1 | 1 | 2 | 1 | 1 | 1 | 1 | 1 | 1 | 5 | 1 | 2 | 2 | 1 | 1 | 1 | 1 | 1 | 1 |
| **7235** | 1 | 1 | 1 | 1 | 1 | 2 | 1 | 1 | 1 | 1 | 1 | 1 | 1 | 1 | 1 | 1 | 1 | 1 | 1 | 1 | 1 | 1 | 1 | 1 | 1 | 1 | 2 | 1 | 1 |
| **7491** | 1 | 7 | 1 | 1 | 1 | 2 | 4 | 5 | 1 | 1 | 1 | 1 | 1 | 1 | 1 | 1 | 2 | 1 | 1 | 3 | 1 | 1 | 1 | 1 | 2 | 1 | 2 | 1 | 3 |
| **7500** | 1 | 2 | 2 | 1 | 1 | 2 | 1 | 5 | 1 | 1 | 1 | 1 | 1 | 1 | 1 | 1 | 3 | 1 | 1 | 5 | 1 | 1 | 1 | 1 | 3 | 1 | 1 | 1 | 1 |
| **7546** | 1 | 2 | 3 | 1 | 1 | 2 | 2 | 6 | 1 | 1 | 3 | 1 | 1 | 1 | 1 | 2 | 3 | 1 | 1 | 5 | 1 | 3 | 3 | 1 | 2 | 3 | 2 | 1 | 2 |
| **7547** | 1 | 4 | 1 | 1 | 1 | 2 | 1 | 4 | 1 | 1 | 1 | 1 | 1 | 1 | 1 | 1 | 2 | 1 | 1 | 4 | 1 | 1 | 1 | 1 | 2 | 1 | 2 | 1 | 3 |
| *Note. The stimulus codes presented in this table were derived from the International Affective Picture System (IAPS). The ratings were collected using the Self-Assessment Manikin (SAM). The letter “S” denotes a subject.* | | | | | | | | | | | | | | | | | | | | | | | | | | | | | |

**Tabe S4** *Valence ratings for each pleasant stimulus as reported by each subject.*

| **Stimuli Codes** | **Subjects** | | | | | | | | | | | | | | | | | | | | | | | | | | | | |
| --- | --- | --- | --- | --- | --- | --- | --- | --- | --- | --- | --- | --- | --- | --- | --- | --- | --- | --- | --- | --- | --- | --- | --- | --- | --- | --- | --- | --- | --- |
|  | **S**  **1** | **S**  **2** | **S**  **3** | **S**  **4** | **S**  **5** | **S**  **6** | **S**  **7** | **S**  **8** | **S**  **9** | **S**  **10** | **S**  **11** | **S**  **12** | **S**  **13** | **S**  **14** | **S**  **15** | **S**  **16** | **S**  **17** | **S**  **18** | **S**  **19** | **S**  **20** | **S**  **21** | **S**  **22** | **S**  **23** | **S**  **24** | **S**  **25** | **S**  **26** | **S**  **27** | **S**  **28** | **S**  **29** |
| **1441** | 6 | 7 | 9 | 7 | 5 | 8 | 7 | 9 | 7 | 7 | 6 | 9 | 9 | 7 | 9 | 7 | 8 | 8 | 9 | 5 | 9 | 8 | 8 | 5 | 7 | 7 | 6 | 7 | 6 |
| **1463** | 6 | 6 | 9 | 8 | 8 | 8 | 8 | 9 | 8 | 7 | 9 | 9 | 8 | 8 | 8 | 9 | 9 | 8 | 9 | 9 | 8 | 9 | 8 | 5 | 8 | 8 | 6 | 9 | 4 |
| **1540** | 5 | 7 | 7 | 7 | 6 | 6 | 7 | 7 | 7 | 7 | 8 | 4 | 6 | 7 | 6 | 6 | 7 | 7 | 9 | 9 | 6 | 6 | 7 | 5 | 8 | 5 | 6 | 8 | 5 |
| **1601** | 7 | 5 | 7 | 8 | 7 | 6 | 8 | 8 | 6 | 8 | 7 | 8 | 7 | 6 | 7 | 7 | 8 | 7 | 8 | 5 | 8 | 7 | 8 | 5 | 6 | 5 | 6 | 9 | 8 |
| **1710** | 6 | 8 | 9 | 7 | 7 | 7 | 8 | 9 | 8 | 7 | 9 | 9 | 8 | 8 | 9 | 9 | 8 | 9 | 9 | 6 | 9 | 7 | 8 | 5 | 7 | 8 | 6 | 9 | 8 |
| **1722** | 5 | 3 | 7 | 7 | 5 | 7 | 6 | 9 | 8 | 8 | 8 | 9 | 8 | 8 | 6 | 9 | 7 | 7 | 9 | 6 | 7 | 8 | 6 | 5 | 8 | 7 | 7 | 6 | 6 |
| **1731** | 8 | 6 | 9 | 8 | 1 | 7 | 6 | 8 | 8 | 8 | 6 | 8 | 7 | 6 | 7 | 7 | 8 | 6 | 9 | 7 | 8 | 8 | 5 | 6 | 6 | 5 | 7 | 2 | 6 |
| **1811** | 6 | 3 | 7 | 7 | 7 | 7 | 8 | 8 | 6 | 8 | 7 | 7 | 7 | 4 | 4 | 6 | 7 | 6 | 6 | 6 | 6 | 7 | 8 | 5 | 3 | 7 | 6 | 5 | 8 |
| **1920** | 5 | 3 | 7 | 8 | 7 | 6 | 8 | 9 | 7 | 5 | 9 | 7 | 7 | 6 | 9 | 8 | 8 | 8 | 7 | 9 | 9 | 8 | 8 | 5 | 5 | 8 | 5 | 9 | 8 |
| **2040** | 6 | 5 | 9 | 7 | 7 | 7 | 7 | 9 | 8 | 8 | 7 | 8 | 7 | 7 | 4 | 6 | 7 | 6 | 8 | 5 | 7 | 8 | 7 | 5 | 5 | 7 | 5 | 9 | 8 |
| **2070** | 6 | 4 | 9 | 7 | 5 | 8 | 7 | 9 | 7 | 8 | 7 | 9 | 7 | 8 | 6 | 9 | 8 | 6 | 8 | 3 | 7 | 8 | 8 | 2 | 6 | 9 | 6 | 7 | 8 |
| **2071** | 5 | 6 | 9 | 7 | 6 | 8 | 6 | 9 | 7 | 9 | 6 | 9 | 7 | 7 | 6 | 8 | 8 | 6 | 8 | 7 | 8 | 7 | 7 | 1 | 6 | 8 | 7 | 7 | 8 |
| **2080** | 5 | 5 | 7 | 7 | 5 | 7 | 6 | 9 | 7 | 4 | 6 | 7 | 6 | 8 | 4 | 7 | 7 | 6 | 8 | 5 | 7 | 6 | 8 | 5 | 5 | 7 | 6 | 5 | 7 |
| **2091** | 7 | 4 | 9 | 8 | 8 | 9 | 7 | 9 | 7 | 9 | 8 | 9 | 7 | 8 | 7 | 9 | 8 | 6 | 8 | 5 | 8 | 6 | 8 | 2 | 7 | 7 | 6 | 8 | 7 |
| **2150** | 7 | 2 | 7 | 7 | 5 | 8 | 6 | 8 | 8 | 8 | 6 | 7 | 1 | 6 | 5 | 7 | 7 | 6 | 6 | 5 | 6 | 8 | 8 | 5 | 5 | 7 | 6 | 8 | 9 |
| **2158** | 7 | 6 | 9 | 8 | 9 | 8 | 7 | 8 | 7 | 8 | 6 | 7 | 6 | 5 | 7 | 9 | 6 | 6 | 7 | 5 | 8 | 6 | 7 | 5 | 6 | 8 | 7 | 8 | 6 |
| **2165** | 6 | 4 | 8 | 8 | 9 | 7 | 6 | 8 | 7 | 9 | 6 | 8 | 3 | 8 | 6 | 6 | 8 | 7 | 6 | 5 | 7 | 7 | 8 | 5 | 6 | 8 | 6 | 8 | 8 |
| **2209** | 6 | 5 | 7 | 7 | 5 | 6 | 7 | 8 | 5 | 8 | 7 | 7 | 7 | 6 | 7 | 6 | 8 | 6 | 5 | 5 | 8 | 7 | 8 | 5 | 7 | 5 | 5 | 6 | 5 |
| **2224** | 6 | 4 | 9 | 7 | 8 | 8 | 6 | 9 | 7 | 8 | 6 | 9 | 6 | 6 | 6 | 6 | 7 | 7 | 8 | 4 | 6 | 6 | 9 | 5 | 6 | 7 | 6 | 8 | 7 |
| **2340** | 6 | 5 | 9 | 7 | 7 | 8 | 7 | 8 | 7 | 8 | 7 | 8 | 4 | 6 | 7 | 6 | 6 | 6 | 7 | 9 | 8 | 7 | 7 | 2 | 6 | 8 | 6 | 9 | 9 |
| **2345** | 6 | 5 | 8 | 7 | 6 | 7 | 7 | 9 | 7 | 8 | 6 | 9 | 6 | 6 | 7 | 7 | 6 | 6 | 8 | 5 | 7 | 8 | 8 | 5 | 6 | 8 | 6 | 9 | 7 |
| **2346** | 2 | 1 | 4 | 6 | 6 | 6 | 4 | 6 | 5 | 7 | 4 | 8 | 3 | 6 | 7 | 5 | 4 | 5 | 6 | 3 | 5 | 7 | 8 | 2 | 6 | 5 | 6 | 8 | 6 |
| **2550** | 5 | 5 | 9 | 8 | 8 | 8 | 7 | 7 | 8 | 9 | 7 | 9 | 6 | 7 | 8 | 6 | 7 | 6 | 6 | 8 | 7 | 6 | 8 | 5 | 6 | 6 | 6 | 7 | 7 |
| **4532** | 5 | 4 | 5 | 7 | 7 | 6 | 7 | 9 | 6 | 6 | 6 | 8 | 7 | 5 | 6 | 5 | 7 | 6 | 5 | 5 | 6 | 6 | 7 | 5 | 6 | 5 | 5 | 7 | 7 |
| **4572** | 5 | 4 | 1 | 7 | 9 | 6 | 5 | 8 | 5 | 8 | 6 | 8 | 6 | 5 | 6 | 5 | 7 | 5 | 6 | 5 | 5 | 8 | 6 | 1 | 7 | 5 | 5 | 6 | 5 |
| **4597** | 6 | 5 | 8 | 7 | 7 | 7 | 6 | 9 | 7 | 7 | 6 | 7 | 7 | 6 | 6 | 5 | 8 | 5 | 5 | 5 | 6 | 7 | 7 | 4 | 6 | 6 | 5 | 6 | 7 |
| **4604** | 7 | 2 | 9 | 7 | 8 | 7 | 8 | 6 | 6 | 8 | 7 | 7 | 9 | 5 | 5 | 5 | 7 | 7 | 5 | 5 | 6 | 7 | 7 | 1 | 6 | 9 | 5 | 6 | 7 |
| **4607** | 7 | 1 | 8 | 7 | 7 | 6 | 9 | 7 | 6 | 8 | 7 | 7 | 8 | 5 | 7 | 5 | 7 | 6 | 6 | 5 | 5 | 7 | 8 | 2 | 6 | 8 | 5 | 6 | 6 |
| **4608** | 6 | 3 | 9 | 7 | 8 | 7 | 9 | 6 | 6 | 8 | 7 | 9 | 8 | 5 | 5 | 5 | 7 | 7 | 5 | 5 | 5 | 8 | 9 | 5 | 6 | 7 | 5 | 7 | 7 |
| **4610** | 7 | 3 | 7 | 7 | 5 | 6 | 6 | 8 | 9 | 5 | 6 | 7 | 7 | 6 | 5 | 5 | 6 | 5 | 1 | 5 | 5 | 7 | 9 | 2 | 7 | 7 | 6 | 7 | 6 |
| **4611** | 7 | 2 | 8 | 7 | 6 | 7 | 9 | 7 | 7 | 8 | 8 | 8 | 8 | 5 | 5 | 5 | 8 | 6 | 5 | 5 | 6 | 8 | 8 | 1 | 6 | 7 | 5 | 6 | 7 |
| **4612** | 6 | 5 | 9 | 7 | 8 | 8 | 8 | 9 | 7 | 7 | 7 | 9 | 7 | 6 | 8 | 5 | 8 | 6 | 6 | 5 | 6 | 7 | 8 | 5 | 6 | 7 | 5 | 7 | 8 |
| **4623** | 7 | 4 | 9 | 7 | 6 | 7 | 8 | 8 | 6 | 8 | 7 | 7 | 7 | 6 | 6 | 6 | 8 | 6 | 5 | 5 | 6 | 7 | 7 | 1 | 6 | 8 | 6 | 8 | 8 |
| **4624** | 7 | 5 | 7 | 7 | 6 | 6 | 6 | 7 | 7 | 8 | 6 | 9 | 7 | 7 | 6 | 5 | 7 | 6 | 5 | 5 | 6 | 6 | 7 | 3 | 6 | 5 | 6 | 7 | 6 |
| **4625** | 6 | 5 | 7 | 7 | 6 | 6 | 7 | 8 | 7 | 8 | 6 | 8 | 6 | 7 | 6 | 6 | 7 | 6 | 6 | 5 | 5 | 7 | 8 | 3 | 5 | 8 | 6 | 6 | 7 |
| **4640** | 7 | 4 | 9 | 7 | 8 | 8 | 7 | 8 | 7 | 7 | 6 | 9 | 7 | 7 | 5 | 5 | 7 | 6 | 5 | 5 | 6 | 6 | 8 | 2 | 6 | 8 | 5 | 7 | 7 |
| **4641** | 6 | 5 | 7 | 7 | 6 | 7 | 8 | 9 | 7 | 7 | 7 | 7 | 7 | 6 | 5 | 5 | 7 | 6 | 5 | 5 | 5 | 7 | 8 | 3 | 6 | 6 | 6 | 8 | 7 |
| **4643** | 7 | 3 | 8 | 7 | 6 | 7 | 8 | 7 | 6 | 8 | 7 | 7 | 7 | 4 | 6 | 5 | 6 | 6 | 5 | 6 | 4 | 7 | 5 | 1 | 6 | 7 | 5 | 9 | 7 |
| **4645** | 6 | 2 | 7 | 7 | 6 | 6 | 7 | 7 | 6 | 7 | 5 | 7 | 7 | 6 | 6 | 5 | 8 | 6 | 5 | 7 | 6 | 6 | 7 | 1 | 5 | 6 | 5 | 7 | 7 |
| **4650** | 7 | 4 | 7 | 6 | 7 | 5 | 6 | 7 | 7 | 8 | 6 | 7 | 6 | 5 | 4 | 3 | 7 | 6 | 5 | 5 | 6 | 7 | 8 | 1 | 5 | 7 | 5 | 8 | 7 |
| **4653** | 7 | 4 | 9 | 7 | 6 | 7 | 9 | 7 | 7 | 7 | 6 | 7 | 7 | 5 | 2 | 6 | 8 | 7 | 5 | 5 | 6 | 7 | 7 | 6 | 6 | 9 | 5 | 7 | 7 |
| **4700** | 6 | 5 | 7 | 7 | 6 | 7 | 6 | 7 | 6 | 8 | 5 | 8 | 7 | 6 | 6 | 6 | 8 | 6 | 6 | 4 | 5 | 7 | 8 | 4 | 6 | 8 | 6 | 8 | 7 |
| *Note. The stimulus codes presented in this table were derived from the International Affective Picture System (IAPS). The ratings were collected using the Self-Assessment Manikin (SAM). The letter “S” denotes a subject.* | | | | | | | | | | | | | | | | | | | | | | | | | | | | | |

**Table S5** *Arousal ratings for each pleasant stimulus as reported by each subject.*

| **Stimuli Codes** | **Subjects** | | | | | | | | | | | | | | | | | | | | | | | | | | | | |
| --- | --- | --- | --- | --- | --- | --- | --- | --- | --- | --- | --- | --- | --- | --- | --- | --- | --- | --- | --- | --- | --- | --- | --- | --- | --- | --- | --- | --- | --- |
|  | **S**  **1** | **S**  **2** | **S**  **3** | **S**  **4** | **S**  **5** | **S**  **6** | **S**  **7** | **S**  **8** | **S**  **9** | **S**  **10** | **S**  **11** | **S**  **12** | **S**  **13** | **S**  **14** | **S**  **15** | **S**  **16** | **S**  **17** | **S**  **18** | **S**  **19** | **S**  **20** | **S**  **21** | **S**  **22** | **S**  **23** | **S**  **24** | **S**  **25** | **S**  **26** | **S**  **27** | **S**  **28** | **S**  **29** |
| **1441** | 2 | 4 | 7 | 1 | 5 | 8 | 3 | 9 | 3 | 2 | 4 | 3 | 5 | 2 | 4 | 4 | 4 | 2 | 1 | 8 | 2 | 3 | 5 | 1 | 3 | 5 | 3 | 8 | 2 |
| **1463** | 4 | 3 | 7 | 4 | 7 | 8 | 4 | 7 | 3 | 2 | 8 | 7 | 4 | 2 | 5 | 6 | 8 | 2 | 1 | 6 | 2 | 3 | 1 | 1 | 3 | 5 | 3 | 9 | 3 |
| **1540** | 1 | 3 | 7 | 2 | 1 | 6 | 2 | 5 | 2 | 2 | 9 | 1 | 2 | 2 | 2 | 2 | 5 | 2 | 1 | 9 | 1 | 2 | 1 | 1 | 2 | 4 | 2 | 8 | 1 |
| **1601** | 3 | 2 | 5 | 2 | 3 | 3 | 3 | 7 | 2 | 2 | 5 | 2 | 3 | 1 | 1 | 4 | 3 | 3 | 1 | 5 | 3 | 2 | 2 | 1 | 2 | 2 | 3 | 9 | 2 |
| **1710** | 6 | 3 | 7 | 3 | 3 | 7 | 3 | 7 | 3 | 2 | 8 | 7 | 3 | 2 | 4 | 6 | 4 | 2 | 1 | 1 | 2 | 4 | 1 | 1 | 2 | 6 | 3 | 9 | 2 |
| **1722** | 2 | 2 | 4 | 2 | 1 | 2 | 3 | 9 | 1 | 2 | 4 | 6 | 3 | 2 | 4 | 4 | 3 | 1 | 1 | 5 | 2 | 2 | 3 | 1 | 4 | 4 | 4 | 2 | 5 |
| **1731** | 6 | 2 | 1 | 1 | 1 | 2 | 2 | 2 | 1 | 2 | 3 | 3 | 2 | 2 | 2 | 4 | 2 | 3 | 1 | 2 | 2 | 4 | 7 | 7 | 3 | 2 | 3 | 4 | 4 |
| **1811** | 3 | 4 | 4 | 2 | 6 | 5 | 3 | 7 | 2 | 2 | 3 | 1 | 3 | 2 | 3 | 2 | 3 | 1 | 1 | 1 | 1 | 5 | 6 | 1 | 3 | 5 | 3 | 1 | 5 |
| **1920** | 3 | 4 | 6 | 2 | 7 | 7 | 3 | 8 | 2 | 3 | 7 | 1 | 3 | 1 | 6 | 4 | 4 | 5 | 1 | 5 | 3 | 2 | 1 | 1 | 3 | 6 | 3 | 9 | 3 |
| **2040** | 3 | 2 | 6 | 3 | 6 | 6 | 2 | 6 | 2 | 2 | 5 | 2 | 2 | 2 | 2 | 2 | 2 | 2 | 1 | 1 | 1 | 2 | 1 | 1 | 2 | 5 | 2 | 9 | 6 |
| **2070** | 4 | 3 | 3 | 3 | 5 | 3 | 2 | 6 | 2 | 4 | 6 | 9 | 3 | 2 | 2 | 4 | 2 | 2 | 1 | 3 | 2 | 5 | 1 | 2 | 2 | 7 | 3 | 7 | 5 |
| **2071** | 3 | 3 | 3 | 4 | 5 | 7 | 2 | 6 | 2 | 4 | 5 | 3 | 3 | 2 | 2 | 4 | 3 | 4 | 1 | 2 | 2 | 3 | 1 | 1 | 2 | 6 | 3 | 7 | 5 |
| **2080** | 2 | 2 | 5 | 3 | 5 | 5 | 3 | 7 | 2 | 2 | 3 | 1 | 2 | 2 | 1 | 3 | 3 | 2 | 1 | 1 | 2 | 2 | 1 | 1 | 2 | 4 | 2 | 5 | 3 |
| **2091** | 6 | 2 | 8 | 5 | 7 | 9 | 3 | 2 | 2 | 3 | 7 | 6 | 3 | 2 | 3 | 5 | 2 | 1 | 1 | 2 | 2 | 3 | 1 | 2 | 2 | 7 | 3 | 8 | 1 |
| **2150** | 5 | 3 | 4 | 3 | 6 | 6 | 3 | 6 | 2 | 2 | 5 | 1 | 8 | 2 | 2 | 2 | 2 | 2 | 1 | 1 | 1 | 3 | 1 | 1 | 2 | 4 | 2 | 8 | 7 |
| **2158** | 6 | 3 | 6 | 5 | 7 | 4 | 3 | 2 | 2 | 2 | 4 | 1 | 2 | 1 | 3 | 4 | 2 | 1 | 1 | 1 | 2 | 2 | 2 | 1 | 3 | 6 | 4 | 8 | 1 |
| **2165** | 7 | 2 | 6 | 3 | 4 | 7 | 2 | 8 | 2 | 5 | 3 | 2 | 4 | 2 | 2 | 2 | 3 | 2 | 1 | 5 | 2 | 3 | 1 | 2 | 3 | 6 | 2 | 8 | 5 |
| **2209** | 3 | 3 | 5 | 2 | 5 | 4 | 3 | 7 | 1 | 2 | 5 | 1 | 2 | 1 | 3 | 3 | 5 | 1 | 1 | 1 | 2 | 2 | 1 | 1 | 2 | 3 | 2 | 5 | 1 |
| **2224** | 4 | 1 | 9 | 5 | 8 | 7 | 2 | 7 | 2 | 2 | 4 | 3 | 2 | 1 | 3 | 3 | 2 | 1 | 1 | 2 | 1 | 1 | 1 | 1 | 3 | 6 | 3 | 8 | 2 |
| **2340** | 5 | 2 | 8 | 3 | 9 | 8 | 3 | 5 | 2 | 2 | 4 | 2 | 4 | 1 | 3 | 2 | 2 | 2 | 1 | 1 | 3 | 2 | 1 | 1 | 2 | 6 | 2 | 9 | 5 |
| **2345** | 4 | 3 | 4 | 6 | 7 | 6 | 3 | 6 | 2 | 2 | 4 | 2 | 2 | 1 | 2 | 2 | 3 | 1 | 1 | 1 | 1 | 2 | 1 | 1 | 2 | 4 | 3 | 9 | 2 |
| **2346** | 2 | 9 | 1 | 4 | 6 | 6 | 2 | 6 | 2 | 2 | 4 | 2 | 4 | 1 | 1 | 2 | 5 | 1 | 1 | 5 | 2 | 3 | 2 | 4 | 3 | 2 | 3 | 8 | 3 |
| **2550** | 5 | 2 | 7 | 2 | 8 | 8 | 3 | 6 | 2 | 4 | 4 | 1 | 2 | 2 | 2 | 2 | 2 | 1 | 1 | 3 | 2 | 2 | 1 | 1 | 2 | 3 | 2 | 7 | 3 |
| **4532** | 2 | 3 | 2 | 3 | 6 | 2 | 3 | 7 | 1 | 2 | 4 | 1 | 3 | 1 | 3 | 2 | 5 | 1 | 1 | 5 | 1 | 1 | 1 | 1 | 2 | 1 | 2 | 7 | 1 |
| **4572** | 1 | 2 | 1 | 2 | 6 | 4 | 1 | 6 | 2 | 3 | 3 | 2 | 4 | 1 | 3 | 3 | 2 | 1 | 1 | 5 | 1 | 3 | 1 | 1 | 4 | 3 | 2 | 6 | 1 |
| **4597** | 6 | 2 | 5 | 3 | 2 | 7 | 2 | 7 | 2 | 2 | 4 | 1 | 4 | 1 | 3 | 2 | 6 | 1 | 1 | 5 | 1 | 3 | 1 | 2 | 2 | 4 | 4 | 6 | 3 |
| **4604** | 7 | 8 | 6 | 5 | 9 | 7 | 7 | 6 | 2 | 3 | 6 | 2 | 7 | 2 | 3 | 2 | 8 | 3 | 1 | 6 | 1 | 7 | 2 | 1 | 3 | 8 | 4 | 8 | 4 |
| **4607** | 6 | 3 | 7 | 3 | 7 | 6 | 9 | 6 | 2 | 3 | 6 | 2 | 4 | 2 | 2 | 2 | 8 | 2 | 1 | 5 | 1 | 5 | 1 | 3 | 3 | 8 | 4 | 7 | 5 |
| **4608** | 7 | 2 | 9 | 4 | 6 | 7 | 7 | 6 | 2 | 3 | 8 | 3 | 6 | 2 | 3 | 3 | 7 | 3 | 1 | 5 | 2 | 5 | 1 | 3 | 3 | 7 | 5 | 8 | 3 |
| **4610** | 7 | 2 | 6 | 3 | 2 | 5 | 2 | 6 | 1 | 2 | 6 | 1 | 5 | 2 | 3 | 2 | 3 | 5 | 2 | 5 | 5 | 3 | 1 | 1 | 3 | 4 | 3 | 5 | 5 |
| **4611** | 8 | 3 | 7 | 6 | 1 | 7 | 8 | 8 | 2 | 4 | 6 | 1 | 7 | 3 | 3 | 2 | 8 | 2 | 1 | 6 | 1 | 3 | 1 | 7 | 3 | 8 | 5 | 8 | 3 |
| **4612** | 6 | 2 | 7 | 1 | 8 | 6 | 5 | 8 | 1 | 2 | 5 | 1 | 3 | 1 | 3 | 1 | 6 | 1 | 1 | 4 | 1 | 3 | 1 | 1 | 3 | 5 | 2 | 7 | 4 |
| **4623** | 8 | 3 | 9 | 5 | 8 | 7 | 7 | 6 | 2 | 2 | 5 | 2 | 3 | 2 | 1 | 2 | 5 | 2 | 1 | 5 | 1 | 4 | 1 | 1 | 2 | 7 | 4 | 8 | 3 |
| **4624** | 6 | 2 | 5 | 3 | 2 | 6 | 1 | 7 | 2 | 3 | 4 | 1 | 3 | 1 | 3 | 2 | 4 | 1 | 1 | 1 | 1 | 2 | 1 | 2 | 3 | 2 | 2 | 7 | 2 |
| **4625** | 7 | 1 | 4 | 4 | 6 | 6 | 4 | 7 | 2 | 2 | 3 | 2 | 2 | 3 | 1 | 2 | 4 | 2 | 1 | 2 | 1 | 2 | 1 | 1 | 2 | 3 | 3 | 6 | 1 |
| **4640** | 7 | 2 | 8 | 3 | 8 | 8 | 3 | 7 | 2 | 3 | 4 | 1 | 3 | 3 | 2 | 2 | 6 | 2 | 1 | 7 | 1 | 3 | 1 | 3 | 2 | 7 | 2 | 7 | 3 |
| **4641** | 7 | 2 | 5 | 4 | 6 | 7 | 6 | 6 | 2 | 1 | 5 | 1 | 2 | 2 | 1 | 1 | 6 | 2 | 1 | 2 | 1 | 4 | 1 | 1 | 2 | 2 | 3 | 8 | 3 |
| **4643** | 7 | 2 | 6 | 6 | 6 | 7 | 7 | 6 | 2 | 5 | 5 | 1 | 3 | 2 | 1 | 2 | 7 | 2 | 1 | 4 | 1 | 2 | 3 | 2 | 3 | 6 | 2 | 8 | 3 |
| **4645** | 7 | 3 | 5 | 6 | 6 | 6 | 3 | 7 | 2 | 3 | 4 | 1 | 3 | 2 | 2 | 2 | 6 | 1 | 1 | 3 | 1 | 2 | 1 | 1 | 2 | 3 | 2 | 7 | 3 |
| **4650** | 7 | 3 | 4 | 4 | 7 | 6 | 5 | 6 | 2 | 4 | 4 | 2 | 3 | 3 | 2 | 2 | 7 | 3 | 1 | 3 | 1 | 3 | 1 | 3 | 2 | 6 | 4 | 8 | 2 |
| **4653** | 6 | 3 | 6 | 6 | 7 | 7 | 9 | 7 | 2 | 2 | 8 | 2 | 3 | 3 | 1 | 2 | 7 | 3 | 1 | 5 | 1 | 3 | 1 | 9 | 3 | 9 | 4 | 9 | 3 |
| **4700** | 6 | 3 | 5 | 2 | 6 | 6 | 3 | 6 | 1 | 3 | 2 | 1 | 2 | 1 | 3 | 2 | 5 | 1 | 1 | 5 | 1 | 3 | 1 | 4 | 2 | 6 | 3 | 8 | 3 |
| *Note. The stimulus codes presented in this table were derived from the International Affective Picture System (IAPS). The ratings were collected using the Self-Assessment Manikin (SAM). The letter “S” denotes a subject.* | | | | | | | | | | | | | | | | | | | | | | | | | | | | | |

**Table S6** *Valence ratings for each unpleasant stimulus as reported by each subject.*

| **Stimuli Codes** | **Subjects** | | | | | | | | | | | | | | | | | | | | | | | | | | | | |
| --- | --- | --- | --- | --- | --- | --- | --- | --- | --- | --- | --- | --- | --- | --- | --- | --- | --- | --- | --- | --- | --- | --- | --- | --- | --- | --- | --- | --- | --- |
|  | **S**  **1** | **S**  **2** | **S**  **3** | **S**  **4** | **S**  **5** | **S**  **6** | **S**  **7** | **S**  **8** | **S**  **9** | **S**  **10** | **S**  **11** | **S**  **12** | **S**  **13** | **S**  **14** | **S**  **15** | **S**  **16** | **S**  **17** | **S**  **18** | **S**  **19** | **S**  **20** | **S**  **21** | **S**  **22** | **S**  **23** | **S**  **24** | **S**  **25** | **S**  **26** | **S**  **27** | **S**  **28** | **S**  **29** |
| **1050** | 4 | 4 | 1 | 5 | 2 | 4 | 2 | 7 | 4 | 5 | 4 | 1 | 4 | 5 | 2 | 3 | 2 | 3 | 5 | 5 | 4 | 4 | 5 | 5 | 4 | 4 | 4 | 1 | 4 |
| **1110** | 3 | 3 | 1 | 5 | 3 | 4 | 3 | 8 | 5 | 5 | 5 | 2 | 5 | 5 | 5 | 3 | 3 | 6 | 5 | 5 | 4 | 4 | 5 | 3 | 4 | 5 | 4 | 2 | 3 |
| **1113** | 3 | 3 | 3 | 4 | 5 | 4 | 3 | 7 | 3 | 5 | 4 | 1 | 4 | 5 | 5 | 2 | 3 | 5 | 5 | 5 | 4 | 5 | 5 | 5 | 4 | 4 | 4 | 1 | 4 |
| **1200** | 4 | 3 | 1 | 5 | 3 | 2 | 2 | 7 | 4 | 5 | 2 | 1 | 5 | 5 | 4 | 4 | 2 | 4 | 5 | 5 | 3 | 2 | 5 | 5 | 4 | 4 | 5 | 1 | 2 |
| **1300** | 2 | 3 | 1 | 4 | 2 | 3 | 4 | 3 | 4 | 4 | 3 | 4 | 5 | 4 | 2 | 5 | 4 | 4 | 5 | 1 | 4 | 3 | 5 | 1 | 3 | 3 | 3 | 1 | 1 |
| **1301** | 4 | 5 | 1 | 5 | 3 | 5 | 5 | 4 | 4 | 5 | 4 | 5 | 6 | 6 | 2 | 5 | 4 | 4 | 5 | 3 | 6 | 4 | 4 | 5 | 4 | 4 | 4 | 3 | 5 |
| **1930** | 3 | 2 | 1 | 4 | 4 | 4 | 4 | 7 | 3 | 5 | 4 | 2 | 4 | 5 | 2 | 8 | 4 | 6 | 5 | 7 | 2 | 3 | 4 | 1 | 2 | 3 | 4 | 1 | 1 |
| **2095** | 4 | 2 | 6 | 3 | 3 | 2 | 1 | 3 | 4 | 2 | 1 | 2 | 2 | 3 | 1 | 1 | 2 | 4 | 5 | 1 | 3 | 3 | 2 | 1 | 3 | 1 | 2 | 1 | 1 |
| **3010** | 2 | 1 | 1 | 1 | 1 | 2 | 1 | 1 | 2 | 1 | 1 | 1 | 1 | 2 | 1 | 1 | 2 | 3 | 5 | 1 | 1 | 2 | 1 | 5 | 1 | 1 | 2 | 1 | 1 |
| **3015** | 1 | 1 | 1 | 1 | 1 | 2 | 2 | 1 | 1 | 1 | 1 | 1 | 1 | 2 | 1 | 1 | 1 | 3 | 4 | 1 | 1 | 2 | 1 | 1 | 1 | 1 | 1 | 1 | 1 |
| **3016** | 2 | 1 | 1 | 2 | 1 | 2 | 1 | 1 | 2 | 2 | 3 | 2 | 1 | 2 | 1 | 1 | 2 | 4 | 4 | 4 | 1 | 2 | 2 | 1 | 2 | 1 | 2 | 1 | 1 |
| **3030** | 1 | 1 | 1 | 2 | 1 | 2 | 2 | 1 | 2 | 2 | 3 | 1 | 1 | 3 | 1 | 9 | 1 | 5 | 5 | 1 | 3 | 3 | 3 | 1 | 2 | 1 | 3 | 1 | 1 |
| **3051** | 1 | 1 | 1 | 1 | 1 | 2 | 1 | 1 | 1 | 2 | 3 | 1 | 1 | 2 | 1 | 1 | 1 | 3 | 4 | 1 | 2 | 1 | 3 | 1 | 2 | 1 | 3 | 1 | 1 |
| **3101** | 2 | 1 | 1 | 1 | 1 | 2 | 2 | 1 | 2 | 2 | 4 | 1 | 1 | 3 | 1 | 9 | 1 | 4 | 4 | 4 | 3 | 2 | 4 | 1 | 2 | 3 | 3 | 1 | 1 |
| **3102** | 1 | 1 | 1 | 1 | 1 | 1 | 1 | 1 | 2 | 1 | 1 | 1 | 1 | 2 | 1 | 1 | 1 | 3 | 4 | 2 | 1 | 1 | 1 | 1 | 1 | 1 | 1 | 1 | 1 |
| **3103** | 2 | 1 | 1 | 3 | 1 | 3 | 2 | 3 | 2 | 4 | 4 | 2 | 1 | 4 | 2 | 1 | 2 | 4 | 5 | 1 | 3 | 3 | 4 | 1 | 2 | 2 | 3 | 1 | 1 |
| **3110** | 2 | 1 | 1 | 2 | 1 | 2 | 1 | 1 | 2 | 1 | 2 | 1 | 1 | 2 | 1 | 1 | 1 | 4 | 4 | 1 | 1 | 2 | 2 | 1 | 1 | 1 | 1 | 1 | 1 |
| **3120** | 1 | 1 | 1 | 1 | 1 | 2 | 1 | 1 | 2 | 1 | 2 | 1 | 1 | 2 | 2 | 1 | 1 | 3 | 5 | 4 | 2 | 2 | 1 | 1 | 1 | 1 | 2 | 1 | 1 |
| **3140** | 1 | 1 | 1 | 1 | 1 | 2 | 1 | 1 | 2 | 2 | 3 | 1 | 1 | 2 | 1 | 1 | 1 | 3 | 4 | 1 | 2 | 1 | 1 | 1 | 1 | 1 | 2 | 1 | 1 |
| **3168** | 2 | 1 | 1 | 1 | 1 | 2 | 2 | 1 | 2 | 1 | 1 | 1 | 1 | 2 | 1 | 1 | 1 | 3 | 5 | 3 | 2 | 1 | 2 | 5 | 1 | 1 | 2 | 1 | 1 |
| **3190** | 1 | 1 | 4 | 4 | 1 | 2 | 7 | 3 | 2 | 5 | 3 | 4 | 2 | 4 | 1 | 5 | 4 | 4 | 5 | 5 | 3 | 5 | 7 | 1 | 2 | 1 | 4 | 1 | 3 |
| **3261** | 1 | 1 | 1 | 1 | 1 | 2 | 1 | 2 | 1 | 1 | 1 | 1 | 1 | 2 | 1 | 1 | 1 | 1 | 4 | 4 | 2 | 1 | 3 | 1 | 1 | 1 | 2 | 1 | 1 |
| **3266** | 1 | 1 | 1 | 1 | 1 | 2 | 1 | 2 | 1 | 2 | 2 | 1 | 1 | 2 | 1 | 1 | 1 | 3 | 4 | 4 | 1 | 2 | 2 | 1 | 1 | 1 | 2 | 1 | 1 |
| **3400** | 1 | 1 | 1 | 1 | 1 | 2 | 1 | 3 | 2 | 1 | 1 | 1 | 1 | 3 | 1 | 1 | 1 | 1 | 4 | 2 | 1 | 2 | 3 | 1 | 1 | 1 | 2 | 1 | 1 |
| **3530** | 2 | 1 | 1 | 2 | 1 | 2 | 2 | 1 | 3 | 3 | 2 | 1 | 1 | 3 | 1 | 1 | 3 | 2 | 4 | 1 | 2 | 5 | 3 | 1 | 3 | 1 | 2 | 1 | 1 |
| **6210** | 5 | 3 | 2 | 3 | 5 | 3 | 4 | 1 | 5 | 5 | 4 | 1 | 4 | 4 | 5 | 1 | 6 | 5 | 4 | 5 | 3 | 5 | 5 | 2 | 2 | 5 | 3 | 2 | 1 |
| **6240** | 5 | 2 | 2 | 3 | 5 | 4 | 4 | 6 | 5 | 5 | 4 | 2 | 3 | 3 | 5 | 3 | 4 | 6 | 4 | 4 | 3 | 6 | 4 | 2 | 3 | 5 | 4 | 1 | 1 |
| **6250** | 5 | 4 | 1 | 2 | 7 | 2 | 1 | 1 | 5 | 4 | 3 | 2 | 3 | 6 | 4 | 1 | 3 | 4 | 5 | 1 | 3 | 2 | 3 | 5 | 3 | 5 | 3 | 1 | 3 |
| **6312** | 3 | 2 | 1 | 2 | 1 | 2 | 3 | 2 | 4 | 4 | 3 | 2 | 1 | 4 | 1 | 1 | 3 | 4 | 4 | 5 | 3 | 4 | 2 | 5 | 3 | 2 | 3 | 1 | 1 |
| **6313** | 3 | 2 | 1 | 3 | 1 | 2 | 1 | 1 | 3 | 3 | 3 | 1 | 1 | 3 | 1 | 9 | 3 | 3 | 5 | 1 | 3 | 5 | 2 | 5 | 2 | 3 | 2 | 1 | 1 |
| **6315** | 3 | 2 | 1 | 2 | 1 | 2 | 3 | 1 | 3 | 4 | 4 | 1 | 1 | 4 | 1 | 9 | 3 | 3 | 4 | 3 | 3 | 6 | 4 | 2 | 1 | 2 | 3 | 1 | 1 |
| **6520** | 2 | 2 | 1 | 2 | 1 | 2 | 3 | 2 | 2 | 4 | 1 | 1 | 1 | 3 | 1 | 2 | 3 | 4 | 4 | 1 | 3 | 2 | 2 | 4 | 3 | 3 | 3 | 1 | 1 |
| **6540** | 3 | 2 | 1 | 2 | 1 | 2 | 3 | 1 | 3 | 4 | 3 | 2 | 1 | 4 | 1 | 9 | 2 | 4 | 4 | 1 | 3 | 2 | 3 | 5 | 3 | 2 | 2 | 1 | 1 |
| **6550** | 2 | 2 | 1 | 2 | 1 | 2 | 2 | 1 | 2 | 4 | 2 | 1 | 2 | 4 | 1 | 1 | 2 | 2 | 4 | 1 | 2 | 5 | 5 | 4 | 6 | 3 | 3 | 1 | 1 |
| **6560** | 3 | 2 | 1 | 3 | 1 | 2 | 3 | 2 | 2 | 4 | 3 | 1 | 1 | 4 | 1 | 1 | 3 | 3 | 5 | 1 | 3 | 7 | 4 | 3 | 7 | 1 | 3 | 1 | 1 |
| **6571** | 5 | 3 | 1 | 2 | 1 | 2 | 3 | 2 | 3 | 5 | 2 | 1 | 3 | 3 | 1 | 9 | 3 | 5 | 4 | 3 | 3 | 5 | 3 | 2 | 7 | 5 | 4 | 1 | 3 |
| **9400** | 1 | 1 | 1 | 1 | 1 | 1 | 1 | 2 | 1 | 3 | 1 | 1 | 1 | 2 | 1 | 1 | 2 | 5 | 1 | 1 | 2 | 3 | 2 | 1 | 2 | 3 | 4 | 1 | 1 |
| **9405** | 1 | 1 | 1 | 1 | 1 | 1 | 1 | 2 | 1 | 1 | 2 | 1 | 2 | 1 | 1 | 1 | 1 | 5 | 2 | 1 | 2 | 4 | 1 | 1 | 8 | 2 | 1 | 1 | 1 |
| **9419** | 2 | 2 | 1 | 2 | 7 | 2 | 8 | 8 | 2 | 3 | 1 | 3 | 2 | 3 | 5 | 2 | 3 | 4 | 4 | 6 | 2 | 2 | 3 | 5 | 3 | 2 | 4 | 1 | 2 |
| **9430** | 5 | 1 | 1 | 2 | 5 | 2 | 2 | 1 | 2 | 3 | 2 | 5 | 1 | 4 | 4 | 9 | 4 | 5 | 4 | 5 | 2 | 2 | 3 | 5 | 3 | 2 | 3 | 1 | 1 |
| **9920** | 3 | 2 | 1 | 2 | 5 | 3 | 3 | 2 | 3 | 3 | 2 | 2 | 1 | 3 | 1 | 2 | 2 | 4 | 4 | 1 | 3 | 4 | 2 | 1 | 3 | 3 | 3 | 1 | 2 |
| **9927** | 4 | 4 | 4 | 5 | 5 | 5 | 2 | 8 | 5 | 4 | 3 | 5 | 5 | 3 | 1 | 5 | 2 | 4 | 5 | 5 | 5 | 4 | 4 | 3 | 3 | 5 | 5 | 5 | 4 |
| *Note. The stimulus codes presented in this table were derived from the International Affective Picture System (IAPS). The ratings were collected using the Self-Assessment Manikin (SAM). The letter “S” denotes a subject.* | | | | | | | | | | | | | | | | | | | | | | | | | | | | | |

**Table S7** *Arousal ratings for each unpleasant stimulus as reported by each subject.*

| **Stimuli Codes** | **Subjects** | | | | | | | | | | | | | | | | | | | | | | | | | | | | |
| --- | --- | --- | --- | --- | --- | --- | --- | --- | --- | --- | --- | --- | --- | --- | --- | --- | --- | --- | --- | --- | --- | --- | --- | --- | --- | --- | --- | --- | --- |
|  | **S**  **1** | **S**  **2** | **S**  **3** | **S**  **4** | **S**  **5** | **S**  **6** | **S**  **7** | **S**  **8** | **S**  **9** | **S**  **10** | **S**  **11** | **S**  **12** | **S**  **13** | **S**  **14** | **S**  **15** | **S**  **16** | **S**  **17** | **S**  **18** | **S**  **19** | **S**  **20** | **S**  **21** | **S**  **22** | **S**  **23** | **S**  **24** | **S**  **25** | **S**  **26** | **S**  **27** | **S**  **28** | **S**  **29** |
| **1050** | 3 | 3 | 8 | 2 | 6 | 6 | 4 | 5 | 2 | 3 | 3 | 9 | 3 | 1 | 4 | 4 | 7 | 3 | 1 | 1 | 2 | 1 | 8 | 1 | 3 | 7 | 2 | 9 | 3 |
| **1110** | 7 | 3 | 4 | 1 | 7 | 3 | 3 | 5 | 1 | 2 | 4 | 2 | 2 | 1 | 2 | 4 | 6 | 2 | 1 | 1 | 1 | 1 | 7 | 1 | 2 | 7 | 2 | 8 | 2 |
| **1113** | 3 | 2 | 5 | 3 | 7 | 4 | 3 | 6 | 2 | 2 | 3 | 4 | 1 | 1 | 1 | 7 | 6 | 1 | 1 | 1 | 1 | 3 | 8 | 1 | 3 | 7 | 2 | 8 | 2 |
| **1200** | 6 | 3 | 5 | 2 | 7 | 3 | 4 | 5 | 2 | 3 | 7 | 9 | 2 | 1 | 2 | 3 | 8 | 3 | 1 | 1 | 2 | 4 | 7 | 1 | 3 | 5 | 3 | 9 | 3 |
| **1300** | 7 | 3 | 7 | 3 | 7 | 6 | 3 | 6 | 2 | 2 | 4 | 2 | 2 | 1 | 3 | 2 | 4 | 2 | 1 | 4 | 2 | 2 | 8 | 8 | 4 | 3 | 2 | 9 | 8 |
| **1301** | 4 | 2 | 6 | 3 | 7 | 3 | 5 | 4 | 2 | 2 | 3 | 1 | 2 | 1 | 1 | 2 | 6 | 2 | 1 | 4 | 1 | 2 | 8 | 1 | 4 | 5 | 2 | 7 | 3 |
| **1930** | 6 | 3 | 9 | 3 | 7 | 3 | 4 | 5 | 5 | 2 | 2 | 2 | 2 | 1 | 2 | 3 | 6 | 2 | 1 | 5 | 3 | 2 | 9 | 3 | 4 | 6 | 2 | 9 | 7 |
| **2095** | 7 | 6 | 4 | 3 | 6 | 5 | 4 | 6 | 2 | 4 | 7 | 4 | 8 | 3 | 1 | 7 | 7 | 1 | 1 | 7 | 3 | 2 | 7 | 7 | 3 | 7 | 3 | 9 | 5 |
| **3010** | 8 | 9 | 9 | 8 | 9 | 7 | 4 | 9 | 7 | 5 | 5 | 9 | 9 | 4 | 5 | 9 | 8 | 4 | 1 | 9 | 4 | 4 | 9 | 1 | 8 | 8 | 4 | 9 | 9 |
| **3015** | 8 | 9 | 7 | 8 | 9 | 8 | 4 | 9 | 6 | 7 | 6 | 9 | 9 | 4 | 3 | 8 | 9 | 2 | 1 | 7 | 4 | 3 | 9 | 9 | 9 | 9 | 3 | 9 | 9 |
| **3016** | 7 | 9 | 7 | 7 | 9 | 8 | 4 | 9 | 8 | 5 | 4 | 3 | 9 | 3 | 3 | 8 | 7 | 2 | 1 | 4 | 3 | 4 | 9 | 9 | 7 | 7 | 3 | 9 | 9 |
| **3030** | 8 | 9 | 9 | 7 | 9 | 8 | 3 | 9 | 6 | 6 | 4 | 6 | 9 | 3 | 2 | 8 | 8 | 1 | 1 | 5 | 3 | 5 | 8 | 8 | 6 | 8 | 3 | 9 | 8 |
| **3051** | 7 | 9 | 7 | 8 | 9 | 8 | 5 | 8 | 7 | 6 | 5 | 9 | 9 | 4 | 5 | 8 | 8 | 3 | 1 | 7 | 4 | 5 | 8 | 7 | 7 | 8 | 2 | 9 | 9 |
| **3101** | 7 | 9 | 8 | 8 | 9 | 6 | 4 | 8 | 5 | 4 | 2 | 8 | 9 | 3 | 6 | 8 | 8 | 2 | 1 | 5 | 2 | 5 | 8 | 7 | 7 | 7 | 2 | 9 | 9 |
| **3102** | 9 | 9 | 8 | 8 | 9 | 8 | 4 | 9 | 7 | 7 | 4 | 9 | 9 | 4 | 7 | 9 | 9 | 3 | 1 | 8 | 4 | 4 | 9 | 8 | 9 | 9 | 3 | 9 | 9 |
| **3103** | 8 | 7 | 6 | 6 | 5 | 6 | 3 | 5 | 6 | 2 | 2 | 4 | 7 | 2 | 3 | 7 | 6 | 2 | 1 | 4 | 2 | 3 | 8 | 9 | 6 | 6 | 2 | 9 | 8 |
| **3110** | 8 | 9 | 6 | 7 | 9 | 7 | 4 | 9 | 8 | 7 | 4 | 7 | 9 | 3 | 7 | 9 | 9 | 1 | 1 | 7 | 4 | 2 | 9 | 9 | 7 | 7 | 4 | 9 | 9 |
| **3120** | 8 | 9 | 6 | 7 | 9 | 8 | 4 | 9 | 8 | 6 | 6 | 8 | 9 | 3 | 4 | 9 | 8 | 2 | 1 | 5 | 3 | 2 | 9 | 9 | 9 | 9 | 3 | 9 | 9 |
| **3140** | 8 | 9 | 7 | 8 | 8 | 8 | 4 | 9 | 5 | 5 | 5 | 9 | 9 | 5 | 7 | 9 | 8 | 2 | 1 | 7 | 3 | 3 | 9 | 9 | 8 | 8 | 3 | 9 | 7 |
| **3168** | 8 | 9 | 8 | 7 | 9 | 9 | 4 | 9 | 7 | 6 | 7 | 9 | 9 | 3 | 7 | 9 | 9 | 1 | 2 | 7 | 3 | 3 | 9 | 1 | 9 | 9 | 3 | 9 | 9 |
| **3190** | 8 | 9 | 3 | 3 | 9 | 2 | 7 | 5 | 3 | 3 | 4 | 1 | 9 | 3 | 5 | 2 | 7 | 2 | 1 | 3 | 1 | 3 | 4 | 9 | 3 | 7 | 4 | 9 | 4 |
| **3261** | 9 | 9 | 1 | 8 | 9 | 8 | 4 | 7 | 7 | 8 | 8 | 9 | 9 | 4 | 8 | 8 | 9 | 6 | 1 | 7 | 4 | 5 | 9 | 9 | 9 | 9 | 3 | 9 | 9 |
| **3266** | 8 | 9 | 9 | 8 | 9 | 8 | 5 | 7 | 6 | 8 | 5 | 9 | 9 | 3 | 6 | 9 | 9 | 2 | 1 | 7 | 4 | 3 | 9 | 9 | 8 | 9 | 3 | 9 | 8 |
| **3400** | 8 | 9 | 9 | 7 | 9 | 9 | 5 | 7 | 6 | 7 | 8 | 9 | 9 | 4 | 5 | 9 | 8 | 4 | 1 | 7 | 3 | 3 | 9 | 9 | 8 | 9 | 3 | 9 | 8 |
| **3530** | 6 | 3 | 7 | 6 | 9 | 6 | 3 | 9 | 4 | 3 | 7 | 9 | 9 | 3 | 4 | 7 | 5 | 3 | 1 | 6 | 3 | 2 | 9 | 6 | 3 | 7 | 3 | 9 | 7 |
| **6210** | 2 | 1 | 5 | 4 | 1 | 6 | 2 | 8 | 2 | 1 | 3 | 4 | 6 | 1 | 3 | 5 | 2 | 2 | 1 | 5 | 3 | 2 | 7 | 2 | 3 | 1 | 3 | 7 | 7 |
| **6240** | 3 | 1 | 4 | 4 | 1 | 5 | 3 | 5 | 1 | 1 | 2 | 2 | 7 | 1 | 3 | 3 | 3 | 3 | 1 | 4 | 2 | 7 | 7 | 4 | 2 | 1 | 4 | 9 | 7 |
| **6250** | 3 | 2 | 9 | 5 | 2 | 6 | 6 | 7 | 1 | 2 | 4 | 3 | 8 | 2 | 4 | 9 | 7 | 3 | 1 | 8 | 3 | 3 | 8 | 1 | 2 | 1 | 2 | 8 | 6 |
| **6312** | 6 | 2 | 6 | 6 | 9 | 6 | 3 | 6 | 2 | 2 | 4 | 4 | 8 | 1 | 5 | 6 | 6 | 2 | 1 | 5 | 2 | 2 | 9 | 2 | 3 | 6 | 3 | 9 | 6 |
| **6313** | 5 | 3 | 4 | 7 | 9 | 7 | 5 | 9 | 3 | 3 | 6 | 3 | 9 | 2 | 5 | 8 | 7 | 2 | 1 | 4 | 2 | 2 | 9 | 1 | 3 | 7 | 3 | 9 | 7 |
| **6315** | 5 | 2 | 9 | 7 | 9 | 8 | 2 | 9 | 2 | 3 | 3 | 6 | 8 | 1 | 4 | 6 | 5 | 4 | 2 | 5 | 3 | 3 | 9 | 2 | 3 | 8 | 4 | 9 | 5 |
| **6520** | 6 | 2 | 9 | 6 | 9 | 6 | 3 | 8 | 5 | 3 | 7 | 9 | 8 | 1 | 4 | 7 | 5 | 2 | 1 | 8 | 2 | 4 | 8 | 2 | 4 | 7 | 2 | 9 | 8 |
| **6540** | 5 | 7 | 9 | 6 | 9 | 8 | 3 | 9 | 2 | 3 | 5 | 2 | 9 | 2 | 6 | 7 | 7 | 2 | 1 | 7 | 2 | 2 | 9 | 2 | 3 | 8 | 3 | 9 | 7 |
| **6550** | 7 | 2 | 9 | 8 | 9 | 8 | 4 | 9 | 2 | 2 | 6 | 9 | 8 | 1 | 6 | 8 | 7 | 3 | 2 | 7 | 2 | 3 | 9 | 3 | 3 | 8 | 4 | 9 | 7 |
| **6560** | 3 | 2 | 9 | 7 | 9 | 8 | 3 | 9 | 2 | 2 | 4 | 8 | 9 | 1 | 6 | 8 | 6 | 3 | 1 | 5 | 3 | 3 | 9 | 4 | 3 | 7 | 4 | 9 | 5 |
| **6571** | 2 | 2 | 9 | 6 | 9 | 9 | 3 | 2 | 3 | 2 | 8 | 5 | 7 | 3 | 3 | 5 | 3 | 2 | 3 | 5 | 2 | 2 | 9 | 2 | 3 | 2 | 4 | 8 | 3 |
| **9400** | 8 | 8 | 9 | 7 | 9 | 7 | 4 | 2 | 7 | 4 | 5 | 9 | 9 | 3 | 3 | 8 | 5 | 3 | 2 | 2 | 5 | 6 | 9 | 8 | 6 | 6 | 5 | 1 | 1 |
| **9405** | 8 | 8 | 9 | 7 | 9 | 8 | 6 | 9 | 8 | 6 | 5 | 9 | 9 | 5 | 3 | 9 | 8 | 3 | 2 | 5 | 5 | 7 | 9 | 6 | 7 | 7 | 6 | 1 | 7 |
| **9419** | 6 | 3 | 6 | 7 | 1 | 8 | 4 | 4 | 6 | 2 | 7 | 2 | 9 | 2 | 2 | 3 | 3 | 1 | 1 | 5 | 3 | 2 | 9 | 1 | 3 | 5 | 2 | 8 | 1 |
| **9430** | 1 | 4 | 6 | 7 | 5 | 6 | 4 | 9 | 7 | 3 | 6 | 1 | 9 | 2 | 1 | 3 | 5 | 2 | 1 | 1 | 2 | 3 | 3 | 1 | 3 | 4 | 3 | 9 | 9 |
| **9920** | 5 | 3 | 7 | 7 | 1 | 5 | 4 | 9 | 2 | 3 | 6 | 3 | 9 | 2 | 2 | 3 | 4 | 1 | 1 | 4 | 2 | 2 | 9 | 9 | 3 | 3 | 2 | 9 | 3 |
| **9927** | 3 | 2 | 3 | 4 | 5 | 2 | 4 | 6 | 1 | 2 | 4 | 1 | 4 | 2 | 2 | 1 | 5 | 1 | 1 | 1 | 2 | 2 | 4 | 3 | 2 | 2 | 2 | 1 | 1 |
| *Note. The stimulus codes presented in this table were derived from the International Affective Picture System (IAPS). The ratings were collected using the Self-Assessment Manikin (SAM). The letter “S” denotes a subject.* | | | | | | | | | | | | | | | | | | | | | | | | | | | | | |

**Table S8** *Mean valence and arousal ratings of stimuli as reported by each subject.*

| **Subjects** | **Stimuli Valence and Arousal** | | | | | |
| --- | --- | --- | --- | --- | --- | --- |
|  | **Neutral**  **Valence** | **Nautral**  **Arousal** | **Pleasant**  **Valence** | **Pleasant**  **Arousal** | **Unpleasant**  **Valence** | **Unpleasant**  **Arousal** |
| **S1** | 5.02 | 1.31 | 6.17 | 4.93 | 2.52 | 6.05 |
| **S2** | 4.05 | 2.33 | 4.26 | 2.81 | 1.88 | 5.33 |
| **S3** | 4.93 | 2.24 | 7.74 | 5.48 | 1.36 | 6.83 |
| **S4** | 5.19 | 1.02 | 7.14 | 2.19 | 2.88 | 2.43 |
| **S5** | 4.5 | 1.1 | 6.55 | 5.5 | 2.17 | 7.33 |
| **S6** | 4.93 | 2.19 | 6.98 | 5.98 | 2.4 | 6.43 |
| **S7** | 5.36 | 1.81 | 7.02 | 3.67 | 2.38 | 3.9 |
| **S8** | 5.86 | 3.62 | 8 | 6.33 | 2.71 | 7.12 |
| **S9** | 5.1 | 1 | 7.55 | 2.6 | 3.12 | 3.79 |
| **S10** | 4.98 | 1.43 | 6.64 | 4.9 | 2.55 | 4.86 |
| **S11** | 5 | 1 | 7.86 | 2.26 | 1.71 | 5.71 |
| **S12** | 5.07 | 1.48 | 6.62 | 3.36 | 2.05 | 7.36 |
| **S13** | 5.05 | 1.05 | 6.21 | 1.79 | 3.29 | 2.38 |
| **S14** | 5 | 1.05 | 6.12 | 2.5 | 1.83 | 3.93 |
| **S15** | 5.07 | 1.24 | 6.24 | 2.71 | 3.19 | 6.36 |
| **S16** | 5 | 1.62 | 7.26 | 4.38 | 2.33 | 6.5 |
| **S17** | 4.83 | 1.19 | 6.31 | 1.95 | 3.79 | 2.31 |
| **S18** | 4.9 | 1 | 6.43 | 1.02 | 4.31 | 1.17 |
| **S19** | 4.71 | 2.57 | 5.55 | 3.67 | 2.79 | 5 |
| **S20** | 5.12 | 1.12 | 6.52 | 1.6 | 2.6 | 2.71 |
| **S21** | 5.31 | 1.24 | 7.07 | 2.93 | 3.17 | 3.12 |
| **S22** | 5.21 | 1.26 | 7.55 | 1.55 | 3.1 | 8.14 |
| **S23** | 4.45 | 1.1 | 3.6 | 1.95 | 2.52 | 4.86 |
| **S24** | 5.48 | 1.69 | 6.05 | 2.52 | 2.74 | 4.88 |
| **S25** | 5.05 | 1.36 | 6.98 | 4.93 | 2.4 | 6.33 |
| **S26** | 4.86 | 1.81 | 5.71 | 2.93 | 2.86 | 2.98 |
| **S27** | 5.45 | 1.38 | 8.6 | 5.33 | 1.57 | 8.48 |
| **S28** | 5.1 | 2.55 | 6.05 | 5.5 | 1.95 | 5.9 |
| **S29** | 5.12 | 1.1 | 7.14 | 3.4 | 2.38 | 5.86 |
| *Note: The ratings were collected using the Self-Assessment Manikin (SAM). The letter “S” denotes a subject.* | | | | | | |

**Table S9.** *Complete summary of statistics from the univariate multilevel model analysis on intervention target, affect, pleasure, enjoyment, and emotional reactivity measures.*

| **Measure** | **Effect** | **df** | ***F*** | **p** | **η_p_^2^** |
| --- | --- | --- | --- | --- | --- |
| HR | Condition | 2,58 | 1274.3 | < .001 | .98 |
| SMS-PA2 | Condition | 2,58 | 30.0 | < .001 | .51 |
|  | Dimension | 1,29 | 17.9 | < .001 | .38 |
|  | Focus | 1,29 | 22.1 | < .001 | .43 |
|  | Condition x Dimension | 2,203 | 4.8 | .009 | .05 |
|  | Condition x Focus | 2,203 | 5.4 | .005 | .05 |
|  | Dimension x Focus | 1,203 | 0.4 | .551 | < .01 |
|  | Condition x Dimension x Focus | 2,203 | 1.5 | .216 | .01 |
| MES | Condition | 2,58 | 3.7 | .030 | .11 |
| FS | Condition | 2,58 | 5.3 | .008 | .15 |
|  | Time | 1,29 | 0.2 | .678 | < .01 |
|  | Condition x Time | 2,58 | 5.1 | .009 | .15 |
| RP | Condition | 2,58 | 10.3 | < .001 | .26 |
| PP | Condition | 2,58 | 18.6 | < .001 | .39 |
| PACES | Condition | 2,58 | 34.8 | < .001 | .55 |
| 400-2000-ms LPP | Condition | 2,58 | 0.2 | .813 | < .01 |
|  | Valence | 2,58 | 59.2 | < .001 | .67 |
|  | Condition x Valence | 4,114 | 2.0 | .100 | .07 |
| 400-2000-ms LPP_diff_ | Condition | 2,57 | < .01 | .99 | < .01 |
|  | Reactivity | 1,29 | 9.8 | .004 | .25 |
|  | Condition x Reactivity | 2,57 | 4.3 | .011 | .13 |
| 400-1200-ms LPP | Condition | 2,57 | 0.4 | .647 | .02 |
|  | Valence | 2,58 | 57.8 | < .001 | .67 |
|  | Condition x Valence | 4,114 | 1.9 | .113 | .06 |
| 400-1200-ms LPP_diff_ | Condition | 2,58 | < .01 | .939 | < .01 |
|  | Reactivity | 1,29 | 23.3 | < .001 | .45 |
|  | Condition x Reactivity | 2,57 | 4.0 | .023 | .12 |
| 1200-2000-ms LPP | Condition | 2,57 | 0.1 | .931 | < .01 |
|  | Valence | 2,58 | 46.9 | < .001 | .62 |
|  | Condition x Valence | 4,114 | 1.6 | .176 | .05 |
| 1200-2000-ms LPP_diff_ | Condition | 2,57 | < .01 | .982 | < .01 |
|  | Reactivity | 1,29 | 1.5 | .231 | .05 |
|  | Condition x Reactivity | 2,57 | 3.8 | .029 | .12 |

HR = Heart Rate. SMS-PA 2 = State Mindfulness Scale for Physical Activity 2. MES = Mental Effort Scale. FS = Feeling Scale. RP = Remembered Pleasure. PP = Predicted Pleasure. PACES = Physical Activity Enjoyment Scale. LPP = Late positive potential. LPP_diff_ = LPP extracted from difference waves between valanced and neutral images.
